# Metal–organic frameworks and their derivatives for metal-ion (Li, Na, K and Zn) hybrid capacitors

**DOI:** 10.1039/d2sc04012c

**Published:** 2022-09-06

**Authors:** Feiyang Zhan, Huayu Wang, Qingqing He, Weili Xu, Jun Chen, Xuehua Ren, Haoyu Wang, Shude Liu, Minsu Han, Yusuke Yamauchi, Lingyun Chen

**Affiliations:** Department of Applied Chemistry, School of Chemistry and Chemical Engineering, Chongqing University Chongqing 401331 P. R. China lychen@cqu.edu.cn; JST-ERATO Yamauchi Materials Space-Tectonics Project and International Center for Materials Nanoarchitectonics, National Institute for Materials Science Tsukuba Ibaraki 305-0044 Japan lsdyy@yonsei.ac.kr y.yamauchi@uq.edu.au; School of Chemical Engineering and Australian Institute for Bioengineering and Nanotechnology, The University of Queensland Brisbane QLD 4072 Australia y.yamauchi@uq.edu.au

## Abstract

Metal-ion hybrid capacitors (MIHCs) hold particular promise for next-generation energy storage technologies, which bridge the gap between the high energy density of conventional batteries and the high power density and long lifespan of supercapacitors (SCs). However, the achieved electrochemical performance of available MIHCs is still far from practical requirements. This is primarily attributed to the mismatch in capacity and reaction kinetics between the cathode and anode. In this regard, metal–organic frameworks (MOFs) and their derivatives offer great opportunities for high-performance MIHCs due to their high specific surface area, high porosity, topological diversity, and designable functional sites. In this review, instead of simply enumerating, we critically summarize the recent progress of MOFs and their derivatives in MIHCs (Li, Na, K, and Zn), while emphasizing the relationship between the structure/composition and electrochemical performance. In addition, existing issues and some representative design strategies are highlighted to inspire breaking through existing limitations. Finally, a brief conclusion and outlook are presented, along with current challenges and future opportunities for MOFs and their derivatives in MIHCs.

## Abstract 2

This review summarizes the progress of metal–organic frameworks and their derivatives as advanced multifunctional platforms for metal-ion hybrid capacitors.

## Graphical abstract

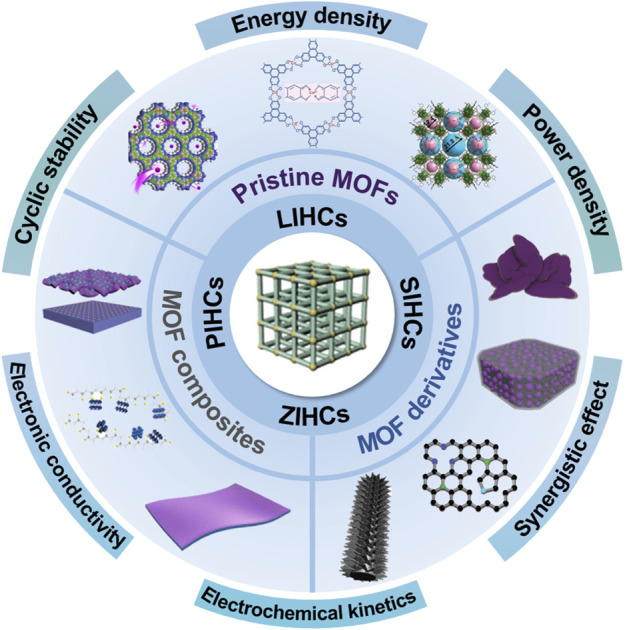

## 1. Introduction

**Fig. 1:**
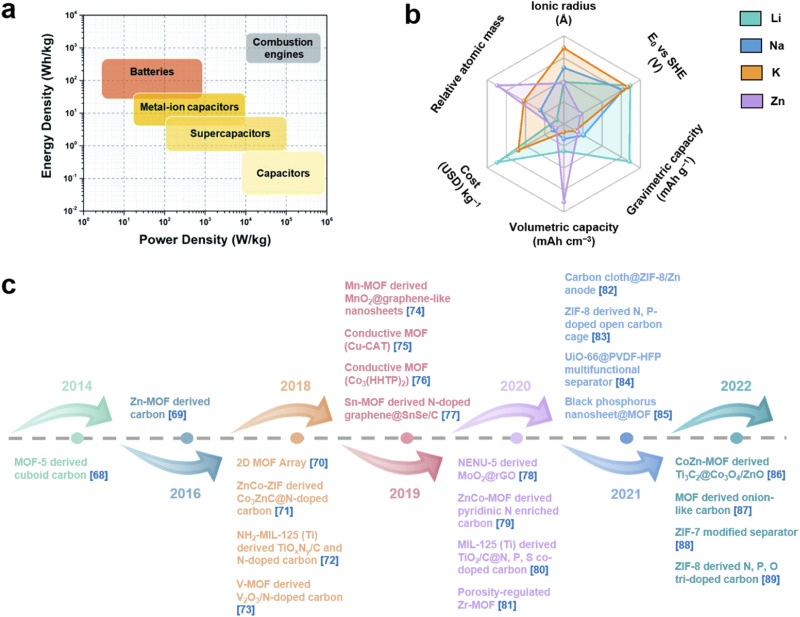
(a) Ragone diagram of different energy storage devices. Reproduced with permission from ref. 42. Copyright 2022, Royal Society of Chemistry. (b) Performance comparison of Li, Na, K and Zn. (c) Recent development of MOFs and MOF derivatives for MIHCs.^68–89^

With the increasing energy consumption, high requirements are put forward for the current energy storage systems,^1,2^ which need to provide high energy density while achieving high power density.^3,4^ As a renewable energy storage alternative, electrochemical energy storage (EES) has received increasing attention.^5,6^ Among them, metal-ion batteries (MIBs) and supercapacitors (SCs) are considered as promising energy and power devices, respectively.^7,8^ MIBs show considerable advantages in energy density through faradaic reactions in active materials, but their power density and cycle life are largely limited due to slow ion diffusion and volumetric strain during the charge–discharge process.^9^ On the other hand, although SCs provide high power density as well as long cycle life through highly reversible storage/release of ions on the electrode surface, their energy density has always been unsatisfactory.^10^ Therefore, it is highly desirable to integrate the advantages of MIBs and SCs into novel energy storage devices. Metal-ion hybrid capacitors (MIHCs) are considered a feasible solution to bridge the gap between MIBs and SCs.^11^ The cathode and anode in MIHCs operate through different mechanisms to simultaneously provide high power and energy densities.^12–15^ As shown in Fig. 1a, the Ragone diagram displays the relationship between energy density and power density for different energy storage devices.

As a hybrid energy storage system, MIHCs integrate the advantages of MIBs and SCs, which offer high energy and long cycle life without sacrificing high power.^16–19^ Lithium-ion hybrid capacitors (LIHCs) were proposed in 2001.^20^ Since then, various MIHCs including sodium-ion hybrid capacitors (SIHCs),^21^ potassium-ion hybrid capacitors (PIHCs),^22^ zinc-ion hybrid capacitors (ZIHCs),^23^ and other types of multivalent MIHCs have been developed.^24–26^ Li, Na, and K are classified as alkali metals, which have low standard hydrogen potentials.^27^ Although Na and K have similar physical and chemical properties to Li, their ionic radius is larger than that of Li^+^, which reduces the reaction kinetics at the electrode/electrolyte interface.^28^ Zn metal has low cost while possessing high stability in aqueous electrolytes. The use of aqueous electrolytes greatly simplifies device assembly, but the performance of current ZIHCs is not yet sufficient for practical requirements.^29^Fig. 1b shows a comparison of the properties of these metals. The typical configurations of MIHCs include a battery-type anode and a capacitor-type cathode. The abundant active sites and faradaic reactions in the anode provide high energy density, while reversible ion adsorption and pseudocapacitive reactions in the cathode are critical for high power density and long operating life.^30,31^ Due to two different charge storage mechanisms, the mismatch in the capacity and kinetics of the anode and cathode makes it difficult to achieve the overall high performance of MIHCs.^32^ A great deal of effort has been made to resolve the imbalance in capacity and kinetics.^33–37^ For battery-type anodes, it is necessary to develop advanced nanostructures that facilitate ion diffusion to improve sluggish kinetics.^38–40^ Meanwhile, electronic conductivity and stability as important indicators also need to be considered.^41^ On the other hand, based on the capacity equation (1/*C*_MIHC_ = 1/*C*_cathode_ + 1/*C*_anode_),^42^ the low specific capacitance of the capacitor-type cathode will lead to a decrease in the specific capacitance of the MIHC device, ultimately resulting in a low energy density. Activated carbon (AC) is commonly used as the cathode material for MIHCs. Despite their large surface area, the lower specific capacitance limits the overall performance of the device.^43^ Therefore, designing novel cathode materials with excellent electrical conductivity, large electrochemically active surface area, and suitable pore distribution is of great significance for MIHCs.^44–46^

Metal–organic frameworks (MOFs) were first defined in 1995 by Yaghi *et al.*^47^ They are crystalline porous materials with intramolecular pores formed by self-assembly of metal ions/clusters and organic ligands.^48–50^ MOFs with desirable structures and functions can be designed by selecting secondary building units constructed from metal ions and rationally designing ligands.^51–53^ MOFs have a large specific surface area and tunable pore size and topology, and their composition determines that they will have some of the properties of both inorganic and organic materials.^54,55^ These properties of MOFs make them show great potential in MIHCs. Furthermore, a composite of MOFs and functional materials will achieve complementary properties.^56^ Among them, the inherent advantages of MOFs are fully utilized, such as porous frameworks and functional molecular/ionic sieves.^57,58^ In another feasible strategy, after thermal/chemical transformation of MOF precursors,^59,60^ the resulting derivatives inherit the advantages of MOFs, such as a large surface area and ideal pore structure, which provide stable electron/ionic transport channels.^61,62^ More importantly, the metal nodes of MOFs are transformed into uniformly distributed metal species, while the organic ligands can be transformed into porous carbons (under inert atmospheres).^63,64^ Combining this strategy with the designability of MOFs will provide advanced functional components for MIHCs. In recent years, studies on MOFs and their derivatives in MIHCs have developed rapidly, which indicates the extension of discoveries of MOFs and their derivatives for application in MIHCs. Although pioneering reviews on MOFs and their derivatives have been proposed,^65–67^ it is still necessary to systematically summarize their notable advances with particular emphasis on MIHCs, including emerging materials, existing issues, device innovations, *etc.*

Here, we provide a comprehensive review of research progress of MOFs and their derivatives in MIHCs, in which representative work is shown in Fig. 1c.^68–89^ We first introduce the construction of MIHCs, including electrode types, cell configurations, and corresponding charge storage mechanisms, and elucidate the properties of the electrolyte. We then outline recent progress by correlating different classes of MOFs/MOF derivatives and electrochemical performance, focusing on the effects of the morphology, composition and structure on electrochemical performance. In view of the existing limitations in electrode materials, several advanced optimization strategies have been proposed to prepare high-performance electrode materials to overcome the mismatch between the cathode and anode. Finally, we discuss existing challenges and potential research directions from multiple perspectives. We hope that this review will deliver an update on the research of MOFs and their derivatives in hybrid energy storage systems and guide future work, thereby inspiring the design and fabrication of novel high-performance MIHCs.

## 2. Construction and mechanistic understanding of MIHCs

As a combination of SCs and MIBs, MIHCs provide a high energy density while maintaining high power characteristics and long cycle life, which are expected to meet the diverse energy storage requirements in the future. MIHCs are composed of electrode materials, current collectors, separators, and electrolytes. Among them, the electrode material is an important factor in determining the performance of MIHCs. The performance of MIHCs, such as power density, rate capability, and cycle life, is mainly determined by the electrode. Common anode materials involve carbon materials, metal oxides, transition metal dichalcogenides, and alloys. For cathode materials, metal oxides and carbon materials are the most common. Furthermore, the matching of battery-type electrodes and capacitor-type electrodes is also critical, and this stems from the kinetic and capacity differences between them. The current collector plays the role of carrying the active material and collecting current. The separator can effectively prevent the cathode and anode from directly coming into contact and causing an internal short circuit. Meanwhile, the electrolyte, as an indispensable component in MIHCs, provides ions during the charging–discharging process and affects the performance of MIHCs, and the operating voltage range of the electrolyte directly affects the energy density of the device. In addition, the interactions between electrodes and electrolytes are complex and important. Herein, we focus on electrode configurations, cell configurations and their associated energy storage mechanisms, as well as electrolytes (aqueous electrolytes, organic electrolytes and ionic liquid electrolytes).

### 2.1. Electrode configurations

Compared with traditional capacitors, MIHCs provide higher energy density due to the large capacity of battery-type electrodes. Meanwhile, high power density can be achieved due to the fast adsorption/desorption process of capacitor-type electrodes. Understanding the mechanisms of cathode and anode materials is of great significance for a comprehensive investigation of MIHC devices. In battery systems, battery-type electrodes provide higher capacity through diffusion-controlled faradaic redox reactions. Meanwhile in SC systems, one mechanism is an electric double-layer capacitor (EDLC)-type mechanism that relies on ion adsorption for charge storage, resulting from the directional arrangement of electrons/ions at the electrode/electrolyte interface, and the other is a pseudocapacitive mechanism utilizing fast surface reversible redox reactions for charge storage.^90^ The electrochemical properties of pseudocapacitive electrodes are neither purely capacitive nor massive faradaic processes, and it is an intermediate state between an EDLC-type mechanism and a battery-type mechanism.^91^ Since the charge storage of SCs is based on the surface reaction of the electrode material, there is no ion diffusion in the electrode material bulk, so the power density is higher than that of the battery, but their energy density is limited.^92^ To improve the energy density of SCs, the construction of asymmetric/hybrid SCs is an efficient strategy, which can increase the overall voltage through the potential difference between the two electrode materials, thus increasing the energy density of SC devices.^93^ Asymmetric SCs (ASCs) cover a wider range of electrode combinations because they can be assembled for SCs that utilize electrodes with the same properties but different mass loads or utilize two electrodes with different materials. Hybrid SCs (HSCs) usually consist of battery-type electrodes and capacitor-type electrodes (different charge storage behaviors),^94^ and such a hybrid device combines the electrochemical properties of SCs and batteries to achieve high power/energy densities. Therefore, HSCs are one of the special cases of ASCs.^95^

**Fig. 2:**
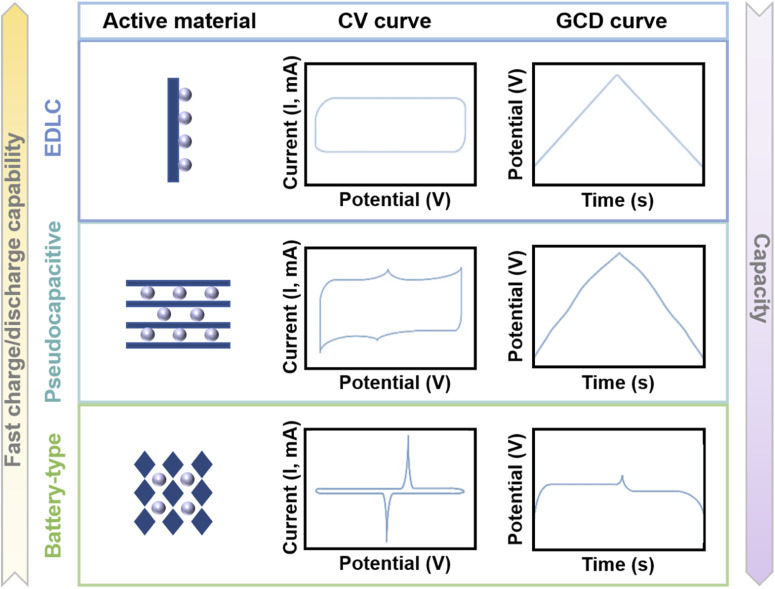
Illustration of the mechanisms, CV curves and GCD curves of EDLC materials, pseudocapacitive materials, and battery-type materials.

Fig. 2 shows the properties of EDLC materials, pseudocapacitive materials, and battery-like materials. The cyclic voltammetry (CV) curve of EDLC materials is a rectangle and the galvanostatic charge/discharge (GCD) curve is a straight line. Meanwhile for pseudocapacitive materials, the CV curve is approximately rectangular, and correspondingly, the GCD curve is approximately linear (compared to EDLCs, the GCD curve can have inflection points, but no obvious platform).^96^ The CV curves of battery-like materials exhibit distinct redox peaks, and the GCD curves provide a clear platform. In addition, the ratio of d*Q*/d*V* is not constant when the electrode material exhibits battery behavior, such as a clear platform in the GCD curve or a clear redox peak in the CV curve. In this case, the capacity should have coulombic (C) or milliampere hour (mA h) as the unit.^90^

**Fig. 3:**
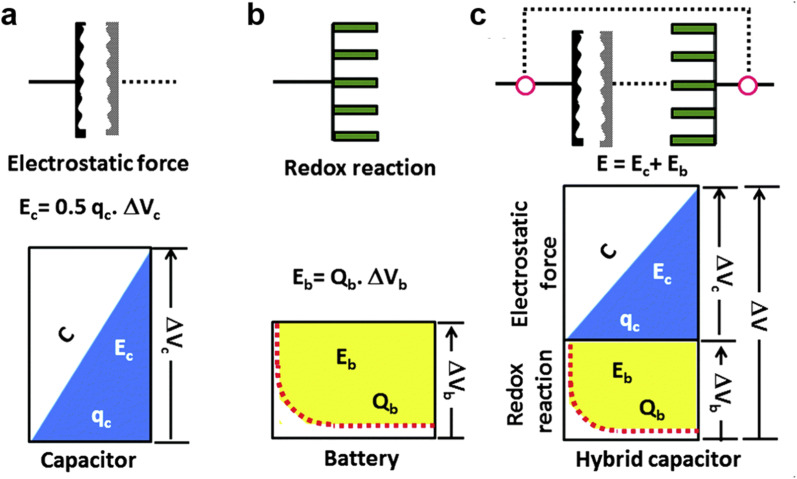
Schematic representations of a single electrode system, (a) capacitor, (b) battery, and (c) hybrid capacitor according to the charge–potential profile with the corresponding equations for energy storage. Reproduced with permission from ref. 97. Copyright 2015, Royal Society of Chemistry.

To gain insight into the electrochemical properties of capacitor-type electrodes and battery-type electrodes and their connection in MIHCs, representative schematic diagrams and charging potential curves are presented in Fig. 3.^97^ As shown in Fig. 3a, the charge–discharge curve of a typical capacitor-type electrode is linear. For a typical battery-type electrode, the potential remains constant during the charging–discharging process according to the phase rule and following the Nernst equation (Fig. 3b). The energy stored in the capacitor can be expressed as *E*_c_, which is lower than the energy of the battery (*E*_b_). If these two systems are effectively combined, the high power characteristics of capacitors and the high energy density of batteries will be integrated into one system (Fig. 3c). In this system, it is essential to utilize a higher working potential (Δ*V*) combined with the capacitor-type electrode at the initial stage of the charging process to reach the redox potential of the battery-type electrode (Δ*V*_b_), thereby increasing the storable amount of energy compared to the single electrode system. However, a significant problem of this hybrid energy storage devices is the mismatch between battery-type electrodes and capacitor-type electrodes. This originates from the sluggish kinetics of battery-type electrodes and the low capacity of capacitor-type electrodes.

Compared with typical bulk battery materials, nanomaterials as electrodes exhibit larger specific surface areas, significantly shortened ion diffusion lengths, and “bulk” redox reactions can be transformed into “surface/near-surface” levels of redox reactions.^98^ Therefore, some battery materials exhibit electrochemical properties similar to pseudocapacitance in the CV and GCD curves, showing significantly accelerated redox reaction kinetics. These rapid redox reaction processes in batteries are not controlled by diffusion due to the nano-size effects.

### 2.2. Cell configurations and charge storage mechanisms

Typically, the configurations of MIHCs generally include a wider range of battery-type anodes and capacitor-type cathodes. Battery-type anodes are generally divided into intercalation-type, conversion-type and alloying-type materials. Additionally, the configuration of a capacitor-type anode and a battery-type cathode is also called the “Upside-Down” configuration.^99^ At this point, the functions of the anode and cathode are reversed from those of the classical device. Battery-type materials operate at higher potentials as a cathode. Due to the potential difference between the anode and cathode, integrated devices with capacitive curves can be obtained. In particular, for zinc-ion energy storage devices, Zn metal can be directly used as an anode,^100^ while the utilization of alkali metals (Li, Na, and K) as an anode is not satisfactory because of dendrite growth. In typical ZIHCs, the configuration is similar to that of zinc-ion batteries except for the cathode material, in which Zn metal acts as the anode and the cathode is usually a carbon material. During the charging–discharging process, energy is stored/released in ZIHCs through rapid ion adsorption/desorption on the surface of the carbon cathode and reversible Zn/Zn^2+^ plating/stripping on the Zn metal anode.^101^ Besides physical adsorption/desorption, chemisorption/desorption of Zn^2+^/H^+^ also occurs. Furthermore, a new type of ZIHC is proposed, usually constructed with battery-type cathodes, capacitor-type anodes, and electrolytes containing zinc salts,^102^ and their charge storage mechanisms are based on Zn^2+^ intercalation/deintercalation on the cathode surface and ion adsorption/desorption on the anode.^103^ Different from the typical carbon-based ZIHCs, the novel ZIHC device avoids the use of unstable Zn foil as an anode. The intercalated anode replaces the traditional Zn foil; however, the intercalated anode needs to have sufficient intercalation spacing of hydrated Zn^2+^, high capacity, appropriate redox potential, and excellent stability, which is beneficial to improve the specific capacity of the device. Meanwhile, such hybrid devices provide a larger voltage window, thus contributing to higher energy density. In fact, with the continuous development of MIHCs, the cell configurations of MIHCs are no longer limited to the typical configuration of a battery-type anode and a capacitor-type cathode. In recent years, different materials have been paired and used to improve the performance of MIHCs. These configurations are gradually deviating from classical MIHCs. Some emerging configuration architectures are gradually being explored. Although various configurations have appeared, cathodes with high specific capacity and anodes with fast kinetics are still regarded as the core of classical MIHCs.^99^

MIHCs can be classified into three types according to the reaction mechanism, including electrolyte consuming, metal-ion exchange, and hybrid mechanisms depending on the consumption of the electrolyte.^104–108^ (1) For the electrolyte consuming mechanism, they are usually based on battery-type materials as an anode and capacitor-type materials as a cathode. During the charge/discharge process, metal cations shuttle between the cathode and anode. In the charging process, metal cations migrate and intercalate into the battery-type anode. Furthermore, the anions in the electrolyte are adsorbed to the surface of the capacitor-type cathode. In the discharging process, metal cations return to the electrolyte from the anode, while anions are desorbed from the cathode to maintain charge balance. (2) For the metal-ion exchange mechanism, they typically include a battery-type material as a cathode to provide a source of metal ions, and a capacitor-type material as an anode. The electrolyte acts only as a transport medium for metal ions and the concentration remains constant in the system. In the charging process, metal cations are deintercalated from the cathode and then into the electrolyte while migrating to the anode surface. In the discharging process, the adsorbed metal cations are desorbed from the anode while entering the electrolyte and further intercalate into the cathode. (3) For the hybrid mechanism, in the charging process, all the metal cations are deintercalated from the cathode and provided by the electrolyte intercalate into the anode. Meanwhile, the cathode absorbs the free anions in the electrolyte. In the discharging process, some of the metal cations are deintercalated from the anode and enter the electrolyte, and the rest of the metal cations are intercalated into the cathode. Moreover, the cathode releases the adsorbed anions into the electrolyte to balance the metal cations in the electrolyte.^109^

### 2.3. Electrolytes

The practical applications of MIHCs require increased energy density without loss of power density, which can be achieved by increasing capacity and enlarging voltage. This mainly depends on the electrode material and electrolyte (including the electrolyte salt and solvent). Among them, the properties of the electrolyte play a key role in the energy/power density, cycle life, and stability of MIHC devices. Factors to consider in electrolyte selection include the potential window, ionic conductivity, stability, flammability, and cost. Commonly used electrolytes can be divided into aqueous electrolytes and nonaqueous electrolytes (organic electrolytes and ionic liquid electrolytes).^110^ Compared with nonaqueous electrolytes, aqueous electrolytes have lower viscosity and a higher ion migration rate. In addition, the safety and low cost of aqueous electrolytes have also attracted more attention to develop aqueous energy storage devices. However, the narrow potential window of aqueous electrolytes limits the wide application of MIHCs. Organic electrolytes can operate at high voltages, but are often toxic and flammable.^111^ Ionic liquid electrolytes have excellent stability and non-flammability;^112^ however, low electrical conductivity and high viscosity limit the performance of MIHC devices. Here, common electrolytes for MIHCs are summarized.

#### 2.3.1. Aqueous electrolytes

Aqueous electrolytes include acidic, alkaline and neutral electrolytes with high ionic conductivity. Among them, neutral electrolytes are more widely used due to their low corrosivity and environmental friendliness, mainly including metal inorganic salt solutions. For neutral electrolytes, achieving high concentrations of electrolytes is significant to ensure high performance.^113^ Neutral aqueous electrolytes such as lithium salt, sodium salt, potassium salt and zinc salt solutions have been investigated.^114^ For aqueous PIHCs, much less work has been reported compared to LIHCs and SIHCs. This is due to the largest ionic radius of K^+^, which leads to irreversible pulverization of the electrode material during the K^+^ intercalation/deintercalation process.^115^ In ZIHCs, cheap and safe aqueous electrolytes represent the majority.^102^ The common ZnSO_4_ electrolyte has low plating/stripping efficiency of Zn and a narrow potential window, which limits the high performance of ZIHCs.^116^ These drawbacks have been ameliorated with the development of Zn(CF_3_SO_3_)_2_ electrolytes.^117^ The formation of by-products during Zn plating is prevented due to the reduced interaction between Zn^2+^ and H_2_O molecules, which is attributed to the interaction of CF_3_SO_3_^−^ and H_2_O. Although aqueous electrolytes offer great convenience for fabricating MIHC devices, the narrow potential window and some undesired side reactions, such as the release of H_2_ or O_2_, limit the high performance of MIHC devices.^100^

#### 2.3.2. Organic electrolytes

Organic electrolytes have wider potential windows than aqueous electrolytes, which can improve the voltage of devices. Organic electrolytes consist of conductive salts and organic solvents, both of them affect the potential window of organic electrolytes, which can provide potential ranges over 4 V, enabling high energy densities.^118^ Meanwhile, the properties of conductive salts and organic solvents, such as ion size, conductivity, ion migration rate, and stability, will significantly affect the device performance. Commonly used organic solvents include dimethyl carbonate (DMC), diethyl carbonate (DEC), ethyl methyl carbonate (EMC), ethylene carbonate (EC) and propylene carbonate (PC).^111^ However, conventional carbonate-based electrolytes usually decompose at a high operating voltage over 4.5 V, and low oxidation potentials may reduce device performance. In general, increasing the concentration of carbonate-based electrolytes promotes the antioxidant properties of the electrolytes. Appropriate electrolyte additives can also maintain high voltage and performance. The emergence of new organic solvents, such as ether-based electrolytes and nitrile electrolytes, has demonstrated the potential to meet high-performance requirements. Other organic electrolytes have been reported for ZIHCs, such as Zn(CF_3_SO_3_)_2_ in acetonitrile (AN).^23^ For the conductive salts of organic electrolytes, the cation-based organic salts are mainly R_4_N^+^, such as Me_4_N^+^, Et_4_N^+^, Et_4_MeN^+^ and Bu_4_N^+^, while the anion-based salts include PF_6_^−^, BF_4_^−^, ClO_4_^−^ and TFSI^−^. In addition, organic electrolytes based on inorganic salts, such as inorganic salts of Li^+^, Na^+^, and K^+^, have also been reported.^11^

#### 2.3.3. Ionic liquid electrolytes

Ionic liquids are organic salts formed by coulombic interactions between cations and charge-delocalized anions, with melting points below 100 °C.^119^ Ionic liquid electrolytes have the advantages of excellent stability, low volatility, and non-flammability. Meanwhile, ionic liquids can not only be used directly as liquid electrolytes, but also as electrolyte salts in organic solvents.^120^ Typical cation-based ionic liquid electrolytes include pyrrolidinium, ammonium and phosphonium. The most commonly used anions of ionic liquid electrolytes are TFSI^−^, NO_3_^−^, PF_6_^−^, BF_4_^−^, Cl^−^, Br^−^ and I^−^.^11^ Despite their numerous advantages, the low ionic conductivity and high viscosity of ionic liquid electrolytes are detrimental to the cycling stability and rate capability of MIHCs. Therefore, it is necessary to optimize ionic liquids with high ionic conductivity and low viscosity.

## 3. Pristine MOFs for MIHCs

By designing the components and structures of MOFs, functional MOFs with high activity and stability can be created for MIHCs. In terms of components, a variety of metal nodes and optional organic ligands can be selected to construct MOFs with various desirable properties. For the structure, the inherently high porosity of MOFs is expected to enable high accessibility of active species and facilitate reaction kinetics due to open pore channels and host–guest interactions.^121^ The construction of MOFs with advanced structures by judicious selection of metal nodes and organic ligands combined with a well-designed pore size/structure provides an outstanding platform for MIHCs.

### 3.1. Role of the conductive framework

Compared with traditional porous materials, MOFs constructed from multifunctional metal ions/clusters and organic ligands have a tunable porosity, composition, and structure. Unfortunately, most MOFs are composed of inactive organic ligands and exhibit low electronic conductivity, which leads to the low utilization of active sites.^122^ In order to improve the conductivity of MOFs, the optimization of ligands and structures is urgently needed.^123^ Representatively, Bao's group synthesized two-dimensional (2D) conductive Co-HAB composed of Co^2+^ nodes and redox-active hexaaminobenzene (HAB) by integrating the high conductivity and high density of redox-active centers.^124^ In an organic electrolyte, the conductive Co-HAB stores nearly three Na^+^ and electrons per HAB, and shows substantial pseudocapacitive contributions, providing an alternative for the construction of high-performance SIHCs.

**Fig. 4:**
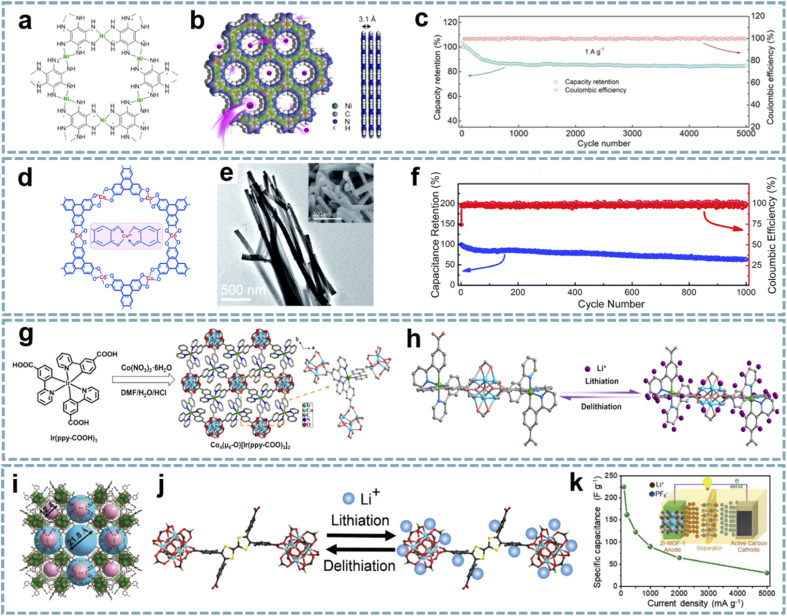
Pristine MOFs for MIHCs. (a) Chemical structure, (b) space-filling model of Ni-HAB. (c) Coulombic efficiency and capacity retention of the SIHC for 5000 cycles. Reproduced with permission from ref. 127. Copyright 2021, American Chemical Society. (d) Chemical structure, (e) TEM image of Co_3_(HHTP)_2_. (f) Cycling stability of the LIHC. Reproduced with permission from ref. 76. Copyright 2019, Royal Society of Chemistry. (g) Synthesis schematic and structural configuration of the Co_4_–Ir MOF. (h) Schematic diagram of the lithium storage mechanism and active sites of the Co_4_–Ir MOF. Reproduced with permission from ref. 128. Copyright 2021, Wiley-VCH. (i) The single-crystal structures of meso–microporous Zr-MOF. (j) Proposed lithiation/delithiation processes in the Zr-MOF anode. (k) Rate performance of the LIHC at different current densities. Reproduced with permission from ref. 81. Copyright 2020, Wiley-VCH.

MOFs with good electrical conductivity as well as rational structures have been developed and used for energy storage devices, which provide a large number of active sites and facilitate the contact of electrode materials with electrolytes.^125^ Conductive MOFs exhibit satisfactory electrochemical performance due to their excellent electron/ion transport properties.^126^ The study of conductive MOFs provides a potential solution for solving the kinetic matching of electrode materials in MIHCs. Dong *et al.* further developed conductive Ni-HAB for fast Na^+^ storage.^127^ The redox active site of Ni-HAB is centered on the HAB linkers, while Ni^2+^ serves as a bridge to stabilize the linker and achieves high electrical conductivity (Fig. 4a and b). Ni-HAB exhibits high rate capability with a capacity of over 100 mA h g^−1^ at a high current density of 10 A g^−1^. The SIHC constructed based on the Ni-HAB anode exhibits high energy/power density and long cycling performance over 5000 cycles (Fig. 4c).

The rational optimization of the morphology can enable faster electron/ion transport. Sun *et al.* reported a one-dimensional (1D) conductive MOF nanowire (Fig. 4d and e),^76^ and the as-synthesized Co_3_(HHTP)_2_ was composed of Co^2+^ and 2,3,6,7,10,11-hexahydroxytriphenylene (HHTP). Each of the ligands can be oxidized to achieve charge balance with the metal centers, which is important for increasing the charge density of Co_3_(HHTP)_2_. As a high-rate anode for LIHCs, Co_3_(HHTP)_2_ with high conductivity provides high reversible lithium storage capacity, exhibiting large capacities of 715 mA h g^−1^ at 0.1 A g^−1^ and 380 mA h g^−1^ at 2 A g^−1^. A full LIHC assembled with Co_3_(HHTP)_2_ and an AC cathode showed a large capacitance of 67 F g^−1^ with a stable operating voltage up to 4 V. However, its cycle life is unsatisfactory (Fig. 4f).

To maximize the exposure of molecular pores to the electrolyte, the synthesis of conductive MOFs on current collectors is an efficient approach. A Cu-based conductive MOF nanowire (Cu-CAT) with a metal catechol (CAT) structure was synthesized on nickel foam.^75^ The synthesis procedure involves a facile solution process of copper acetate and HHTP. The effective orbital overlaps between Cu^2+^ and organic ligands endow Cu-CAT with remarkable charge transport properties. Benefiting from the uniform molecular pore structure, Cu-CAT exhibits great potential as an anode for SIHCs. The process of sodium storage is through the coordination of Na^+^ with O in the C–O bond, accompanied by the reduction of Cu^2+^ to Cu^+^.

The design of MOFs with bi-continuous electronic/ionic conducting networks will greatly improve electrochemical performance. Rationally tailoring the topological structures and components of MOFs is a promising route to realize high electrical conductivity and energy density. A cluster-bridging-coordinated bimetallic Co_4_–Ir MOF has been designed as a high-rate anode for LIHCs (Fig. 4g).^128^ The Co_4_–Ir MOF is assembled from Co_4_(μ_4_-O) clusters bridge-coordinated by Ir(ppy-COOH)_3_, while its ordered porous framework and laminated stacking structure provide favorable conditions for the rapid transport of Li^+^ (Fig. 4h). This bimetallic conductive MOF anode can be well matched with capacitor-type cathodes when applied to LIHCs.

Despite the tremendous progress in research on conductive MOFs as electrodes for MIHCs, their practical application is still in its infancy. Moreover, the specific surface area and functionality of current conductive MOFs cannot meet practical demands. On the other hand, their ligands are generally expensive, which seriously hinders their commercial application. Therefore, the design and optimization of ligands will be of great value in the future.

### 3.2. Role of the open framework

The open framework structure offers multiple advantages, such as fast kinetics and robust durability. Prussian blue analogues (PBAs) can be regarded as the simplest MOFs. As a class of PBAs, metal hexacyanoferrates (M-HCF) have open frameworks and multiple active sites.^129^ Their larger interstitial spaces facilitate the intercalation/deintercalation of metal ions in the cyano-bridged network.^130^ In Pazhamalai's research,^131^ aqueous LIHCs based on Cu-HCF and graphitic carbon were fabricated. Cu-HCF and graphitic carbon were used as an intercalation electrode and a capacitor electrode, respectively. Electrochemical studies demonstrate the existence of intercalation capacitance in Cu-HCF, which stores charges through the intercalation/deintercalation of electrolyte ions. The constructed aqueous LIHC with a potential window of 2.2 V exhibits a high specific capacitance of 63.64 F g^−1^ and a high energy density of 42.78 W h kg^−1^, as well as a long cycle life. Although MOFs with open frameworks have shown potential applications in MIHCs, their capacities are still unsatisfactory. The optimization of the structure and the modulation of the active site are expected to pave the way for their further applications.

### 3.3. Role of porosity

The reticular chemistry of MOFs ensures ordered porosity and abundant active sites.^132^ Meanwhile, the regulation of the assembled form and length of ligands can effectively adjust the microenvironment of pores in MOFs.^133,134^ Appropriate micro/mesoporous channels can provide pathways for the rapid transport of ions.^135^ MOFs with redox-active metal centers, or organic ligands capable of faradaic redox reactions, offer potential alternatives for fabricating high-performance electrodes in MIHCs.^136^ Yan *et al.* reported Zr-MOFs with high crystallinity and high stability composed of Zr^4+^ and tetrathiafulvalene (TTF) based ligands (Fig. 4i).^81^ The micro/mesoporous channels of Zr-MOFs are favorable for ion transport, and the hierarchically porous structure facilitates the rapid diffusion of Li^+^ during the charging–discharging process (Fig. 4j). Meanwhile, the excellent electrochemical redox activity based on TTF ligands ensures fast electrochemical kinetics. With Zr-MOF as a pseudocapacitive anode, matched with an AC cathode, the fabricated LIHC exhibits a high energy density of 122.5 W h kg^−1^ and a high power density of 12.5 kW kg^−1^ in the operating voltage range of 1.0–4.0 V (Fig. 4k).

### 3.4. Summary

2D conductive MOFs have graphitic-like structures and form 2D layer lattices through planar coordination between metal nodes and organic ligands. Furthermore, these 2D layers are stacked along the *z*-axis *via* π–π interactions, resulting in regular and open channels that provide shortened paths for fast ion diffusion. The 2D structure provides an ultra-high specific surface area with abundant active sites, which is beneficial for ion adsorption, surface redox reactions, and ion storage processes. The rational optimization of the morphology can enable fast electron/ion transport. Meanwhile, the synthesis of MOFs on the substrate is beneficial to expose more molecular pores, which originate from the directional growth of MOFs. The rational modulation of the composition and topology of MOFs is a promising approach to achieving high electrical conductivity and energy density, and bimetallic MOFs can provide more active sites for better electrochemical performance.

The open frameworks of PBAs contain open channels and gap sizes, which provide abundant 3D diffusion channels for the diffusion of various charge carrier ions, and the robust open framework structure leads to good cycling stability of PBA-based electrodes. PBAs exhibit excellent electrochemical performance in aqueous devices. In addition to the traditional intercalation/extraction of charge carrier ions, the co-intercalation/extraction of protons and metal ions is observed. For the intercalation of multivalent metal ions in PBA electrodes, the poor diffusion kinetics is mainly due to the large electrostatic interaction between the multivalent metal ions and the host framework. Structural water can accelerate the diffusion of multivalent metal ions through a charge shielding mechanism. However, the presence of structural water promotes the formation of crystal vacancies in PBAs and leads to a shortened cycle life of the electrodes. Therefore, the balance between the structural water content and cycle life of PBA electrodes needs to be explored. Pristine MOFs as electrode materials for MIHCs are summarized in Table 1.

**Table 1:** Pristine MOFs as electrode materials for MIHCs

MOFs	Applications	Configurations	Energy density (W h kg^−1^)	Power density (W kg^−1^)	Cycling stability	Ref.
Ni-HAB	SIHC	Ni-HAB//Na_3_V_2_O_2_(PO_4_)_2_F/AC	127	17 309	5000 cycles (1 A g^−1^)	127
Cu-CAT	SIHC	Cu-CAT//AC	132.85	2980	100 cycles	75
Co_3_(HHTP)_2_	LIHC	Co_3_(HHTP)_2_//AC spheres	64	10 000	1000 cycles (1 A g^−1^)	76
Co_4_–Ir MOF	LIHC	Co_4_–Ir MOF//AC	165.4	12 000	3000 cycles (4 A g^−1^)	128
Cu-HCF	LIHC	Cu-HCF//graphitic carbon	42.78	2619	5000 cycles (5 mA cm^−2^)	131
Zr-MOF	LIHC	Zr-MOF//AC	122.5	12 500	1000 cycles (2 A g^−1^)	81

## 4. MOF composites for MIHCs

Due to the low electrical conductivity of most pristine MOFs, compounding with conductive materials can effectively ameliorate this shortcoming. Meanwhile, MOFs can also be used as supports to load active substances, and their structural stability provides more possibilities to achieve enhanced performance.^137^ On the other hand, the synergistic effect exhibited by MOFs combined with active materials can provide excellent electrochemical performance and special functions. Typically, carbon materials, metal species, active materials, conductive substrates, and polymers are selected for compounding with MOFs.^138^

### 4.1. MOF modified electrode materials

**Fig. 5:**
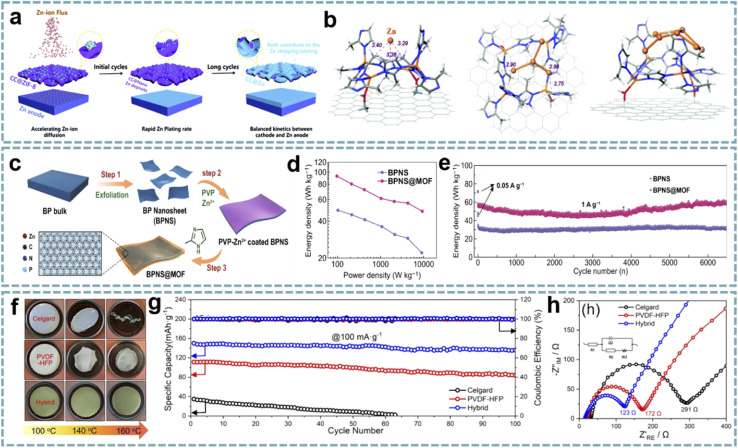
MOF composites for MIHCs. (a) The “nucleation-growth-cycling” process of CC@ZIF-8 combined with a Zn anode. (b) DFT optimized positions of 1, 4 or 7 Zn atoms adsorbed on CC@ZIF-8. Reproduced with permission from ref. 82. Copyright 2021, Royal Society of Chemistry. (c) Schematic diagram of the synthesis process of BPNS@MOF. (d) Ragone plots of BPNS@MOF//AC and BPNS//AC for PIHCs. (e) Cycling performance of BPNS@MOF//AC and BPNS//AC for PIHCs. Reproduced with permission from ref. 85. Copyright 2021, Springer. (f) Thermal shrinkage test of the hybrid separators, PVDF-HFP, and Celgard. (g) Cycling performance and (h) Nyquist plots of the hybrid separators, PVDF-HFP, and Celgard. Reproduced with permission from ref. 84. Copyright 2021, Elsevier.

MOFs as metal ion modulation layers can effectively regulate the deposition behavior of metals.^139,140^ Therefore, MOF-based interface engineering to optimize the stability and reaction kinetics of electrode materials has attracted considerable attention. The surface modification of Zn anodes in ZIHCs provides an advanced strategy for fabricating high-performance Zn-based energy storage devices. Among them, modulating the diffusion, nucleation, and deposition behaviors of Zn^2+^ through MOF composites to balance the kinetic differences between the anode and cathode in ZIHCs has brought light to this research direction. In typical ZIHCs, the slow stripping/plating rates of Zn lead to dendrite growth and accumulation of ions at the electrode/electrolyte interface, thus creating concentration polarization. Leng *et al.* achieved fast and uniform Zn deposition by pre-growing ZIF-8 (ZIF stands for zeolitic imidazolate framework) on carbon cloth (CC@ZIF-8),^82^ and CC@ZIF-8 accelerated the diffusion rate of Zn^2+^ on the Zn anode (Fig. 5a). The nano-Zn deposits induced by ZIF-8 act as Zn nuclei, while the CC with uniform Zn deposition acts as an ion supplementary layer to enhance the stripping/plating rate of Zn (Fig. 5b). The assembled ZIHC provides a high specific capacitance (302 F g^−1^ at 0.5 A g^−1^), a high rate capability (188 F g^−1^ at 20 A g^−1^), and a cycle life of over 10 000 cycles at 1 A g^−1^ (100% capacitance retention).

A similar strategy was extended to PIHCs. An ultrathin ZIF-8 interphase layer with ordered pores and high stability was constructed on the surface of black phosphorus nanosheets (BPNSs) (Fig. 5c).^85^ The as-prepared BPNS@ZIF composites showed superior performance to BPNSs when used as the anode for PIHCs (Fig. 5d and e). This is attributed to the ordered porous structure and high stability of the ZIF interfacial layer, which are important factors that facilitate the diffusion of K^+^ and accommodate the volume change of black phosphorus during the charging–discharging process.

Due to the ordered pore structure and inorganic–organic hybrid properties of MOFs, their use as modulation layers on the surface of electrode materials can achieve high durability and uniform deposition of metal ions. The functionalities of MIHC electrode materials can be improved by the flexible design of the pore structure and framework chemistry of MOFs.

### 4.2. MOF modified separators

Most explorations of MOFs and their derivatives have focused on designing or optimizing electrode materials, but few have focused on multifunctional separators. The inorganic–organic hybrid properties and pore structure of MOFs offer several advantages for the modification of separators, especially efficient molecular/ion sieving capabilities.^141^ MOFs with a tunable porosity, ordered pore structure, and large surface area are used for the separation of soluble mediators and gas adsorption/separation. In addition, by appropriately adjusting the pore size, the target ions can be separated from the solution.

Polymers can be used both as conductive and protective components of electrode materials and as functional separators for energy storage devices.^142,143^ The development of composites of MOFs and polymer separators is of great significance for energy technology. In Feng's report,^84^ activated UiO-66 (UiO stands for the University of Oslo) was selected as the functional active filler to modify a porous poly(vinylidene fluoride-hexafluoropropylene) (PVDF-HFP) separator. Among them, the activated UiO-66 has open pores to absorb more electrolyte ions, and the acid defect sites are conducive to the transport of Na^+^. More importantly, UiO-66 does not react with Na^+^, effectively avoiding the dendrite growth. The excellent mechanical strength and thermal stability of the hybrid separator ensure the safety of SIHCs (Fig. 5f–h). A quasi-solid-state SIHC based on the multifunctional separator offers a high energy density (182 W h kg^−1^ at 31 W kg^−1^), a power density (5280 W kg^−1^ at 22 W h kg^−1^), and long-term cycling stability (10 000 cycles at 1 A g^−1^).

On the other hand, based on the structural properties of MOFs, the composite of MOFs with commercial separators provides a feasible route to achieve durable and well-performing MIHCs. In a recent report, ZIF-7 was used in the modification of glass fiber separators to suppress the shuttle effect of soluble redox mediators.^88^ This confinement effect comes from the physical obstruction and chemisorption of ZIF-7, the micropores of ZIF-7 restrict the diffusion of soluble redox mediators, and the chemisorption prevents their intergranular migration. This synergy allows LIHCs to display high energy density and long-term cycling stability.

### 4.3. Summary

MOFs as metal ion modulation layers effectively adjust the deposition behavior of metals, which originates from the ordered pore structure and inorganic–organic hybrid properties of MOFs. In particular, Zn metal is widely used as the anode of ZIHCs. The surface modulation of the Zn anode with MOFs can regulate the diffusion and deposition behavior of Zn^2+^ to balance the kinetic differences between the anode and cathode in ZIHCs. Although metal anodes are not suitable for LIHCs, SIHCs and PIHCs, the ordered pore structure and high stability of MOFs enrich the functionalities of electrode materials and accommodate the volume changes of electrodes during charging and discharging. On the other hand, the inorganic–organic hybrid properties and pore structure of MOFs provide efficient molecular/ionic sieving ability for the modification of separators. The pore structure of MOFs can confine the diffusion of soluble mediators and provide chemisorption. By adjusting the pore size, the sieving of target ions can also be achieved. MOF composites as functional components for MIHCs are summarized in Table 2.

**Table 2:** MOF composites as functional components for MIHCs

MOF composites	Applications	Configurations	Energy density (W h kg^−1^)	Power density (W kg^−1^)	Cycling stability	Ref
**MOF modified electrode materials**						
Carbon cloth@ZIF-8	ZIHC	Zn/carbon cloth@ZIF-8//carbon	107.4	16 200	10 000 cycles (1 A g^−1^)	82
Black phosphorus@ZIF-8	PIHC	Black phosphorus@ZIF-8//AC	93	9380	6500 cycles (1 A g^−1^)	85

**MOF modified separators**						
UiO-66@PVDF-HFP	SIHC	TiO_2_//AC	182	5280	10 000 cycles (1 A g^−1^)	84
Glass fiber@ZIF-7	LIHC	Li_2_TiSiO_5_//AC	238.56	14 295	4000 cycles (2 A g^−1^)	88

## 5. MOF derivatives for MIHCs

Due to the limited stability and electrical conductivity of pristine MOFs, MOF derivatives have been widely explored for EES.^144^ MOFs with a tunable composition and structure can serve as ideal templates for constructing nanostructures. The inherent organic ligands in MOFs provide a functional platform for their annealing in an inert atmosphere to form carbon materials. Importantly, the inorganic–organic hybrid structure of MOFs easily achieves uniform distribution of metal components and carbon.^145^ Porous structures can be achieved with the decomposition of organic ligands and evaporation of metals at high temperatures. Meanwhile, the pore size of the derivatives can be tuned by designing the pore structure of the MOF or controlling the conditions of pyrolysis. The control of pore size and efficient combination of pores with different sizes would be beneficial for fast electron and mass transport. For the functionalization of MOF derivatives, for example, the introduction of heteroatoms into nanostructures can provide improved conductivity and more active sites. Among them, some ligands containing heteroatoms can easily achieve heteroatom doping after heat treatment.

Generally, the calcination of MOFs in air is a common strategy to convert them into metal oxides. Annealing MOFs in an inert atmosphere reduces the metal species to metal nanoparticles, while the organic ligands decompose and transform into porous carbon. In this case, the metal species will convert to a metal oxide if a source of oxygen (such as oxygen in the ligand) is present. Annealing MOFs in the presence of sulfur, selenium, or phosphorus sources in an inert atmosphere will produce sulfides, selenides, and phosphides, respectively.^146^ The fabrication of metal-free porous carbons is often performed by removing metal species through acid solutions or by evaporating low-boiling metals at high temperatures. In some cases, MOFs can be converted into porous metal compounds by hydrothermal methods.^147^

### 5.1. MOF-derived metal compounds

**Fig. 6:**
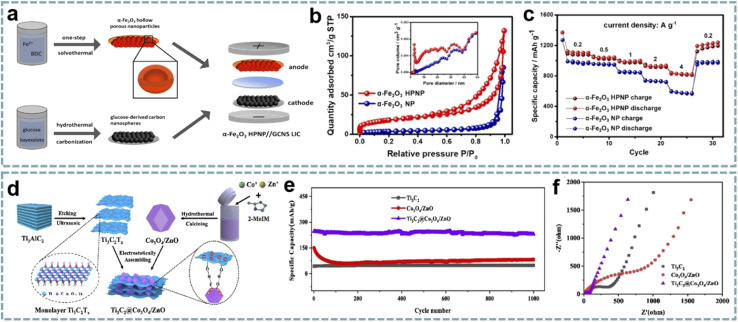
MOF-derived metal compounds for MIHCs. (a) Schematic diagram of the fabrication of the α-Fe_2_O_3_ HPNP//GCNS LIHC. (b) N_2_ adsorption/desorption isotherms and pore size distributions of the α-Fe_2_O_3_ NP and α-Fe_2_O_3_ HPNP. (c) Specific capacities of the α-Fe_2_O_3_ NP and α-Fe_2_O_3_ HPNP at increasing current densities. Reproduced with permission from ref. 149. Copyright 2021, American Chemical Society. (d) Schematic diagram of the fabrication of Ti_3_C_2_@Co_3_O_4_/ZnO. (e) Cycling performance of Co_3_O_4_/ZnO, Ti_3_C_2_, and Ti_3_C_2_@Co_3_O_4_/ZnO over 1200 cycles at 2 A g^−1^ in a Li-half cell. (f) Nyquist plots of Co_3_O_4_/ZnO, Ti_3_C_2_, and Ti_3_C_2_@Co_3_O_4_/ZnO. Reproduced with permission from ref. 86. Copyright 2022, Elsevier.

The unique structure, high porosity and large specific surface area of MOFs make them excellent templates for the preparation of different nanomaterials. Using MOFs as templates to obtain metal compounds has achieved a higher surface area and uniform distribution of active materials.^148^ Furthermore, developing advanced nanoarchitectures based on the structural advantages of MOFs can not only shorten the diffusion paths of ions to optimize reaction kinetics, but also effectively alleviate the volume change of battery-type materials during charging and discharging. Tan *et al.* demonstrated that hollow porous α-Fe_2_O_3_ nanoparticles (α-Fe_2_O_3_ HPNPs) are high-performance anodes for LIHCs.^149^ Among them, the material and nanostructure of the electrode are both critical. The conversion of Fe-MOFs to α-Fe_2_O_3_ HPNPs was achieved by a one-step solvothermal method (Fig. 6a). The porous structure of α-Fe_2_O_3_ HPNPs shortens the diffusion path of Li^+^ and accelerates the diffusion of Li^+^ (Fig. 6b and c), and the hollow structure buffers the volume change of α-Fe_2_O_3_ during the cycling process. When α-Fe_2_O_3_ HPNPs were paired with a high specific surface area carbon nanosphere cathode and a LIHC was assembled, the device provided a high energy density of 107 W h kg^−1^ at a power density of 0.24 kW kg^−1^, and maintained an energy density of 86 W h kg^−1^ at a high power density of 9.68 kW kg^−1^. It also exhibits excellent cycling stability, achieving a high capacity retention of 84% after 2500 cycles at 1 A g^−1^.

In another report, a composite of bimetallic MOF-derived Co_3_O_4_/ZnO with Ti_3_C_2_ nanosheets was achieved by electrostatic assembly (Fig. 6d).^86^ The hollow porous Co_3_O_4_/ZnO provides abundant active sites and open channels for lithium storage, while the Ti_3_C_2_ conductive network provides fast electron/ion transport (Fig. 6e and f). This advanced hybrid structure shows excellent structural stability, thereby enhancing the electrochemical performance of the LIHC. When matched with an AC cathode, the LIHC delivered a high energy density of 196.8 W h kg^−1^ at a power density of 175 W kg^−1^.

The MOF-derived metal compounds achieve a uniform distribution of active species, which is attributed to the ordered metal nodes in MOFs. Although the controlled synthesis and structural design of MOF precursors can provide more active sites and fast kinetics, their electrical conductivity still needs to be improved, and the volume expansion during conversion reactions and alloying needs to be addressed. Therefore, MOF-derived metal compounds/carbons seem to be more popular because of their improved electrical conductivity and diverse functionalities.

### 5.2. MOF-derived carbons

Carbon materials have good electronic conductivity and chemical stability, making them competitive candidates for energy storage systems. In particular, optimized carbon materials with reasonable pore size distribution or heteroatom doping tend to provide satisfactory performance. Generally, carbon materials are prepared by the carbonization of organics. However, their porous structure and chemical composition are difficult to customize.^150^

MOFs are porous polymers assembled from metal ions/clusters and organic ligands. Compared with traditional precursors for carbon materials, MOFs have become a popular choice due to their customizable composition, pore structure, and ease of functionalization with heteroatoms.^151^ These advantages make them ideal templates for the fabrication of porous carbons. Due to the organic components in MOFs, porous carbons can be easily fabricated by the pyrolysis of MOFs without additional carbon sources.^152^ Meanwhile, these advantages have encouraged researchers to explore MOF-derived carbons in energy storage systems, especially MIHCs. Banerjee and co-workers reported a high surface area 3D carbon cuboid cathode fabricated from MOF-5, and paired with a Li_4_Ti_5_O_12_ anode in a LIHC.^68^ Gu *et al.* prepared a high-performance SIHC with ZIF-8-derived carbon as a capacitor-type anode and P2-Na_0.67_Co_0.5_Mn_0.5_O_2_ as a battery-type cathode, achieving high power and energy density.^153^ Interestingly, the pore size, heteroatom doping, and functional groups of MOF-derived carbons provide structural and compositional advantages for the construction of high-performance electrode materials.

#### 5.2.1. Role of pore size

In typical carbon materials, such as widely used AC, despite their large specific surface area, their narrow microporosity leads to inefficient storage of solvated ions.^154^ However, the highest capacitance is expected when the pore size of the carbon matches the size of the solvated ions.^155^ The pore structure significantly affects the accessibility and storage capacity of solvated ions in the electrolyte. In general, macropores provide a shortened pathway for the rapid transport of electrolytes, while micropores and mesopores provide larger surface areas to fully expose more accessible active sites.^156,157^ The hierarchical pore structure of porous carbon can not only provide abundant reaction sites during electrochemical reactions, but also satisfy ion transfer and ion storage during fast adsorption/desorption.^158^

**Fig. 7:**
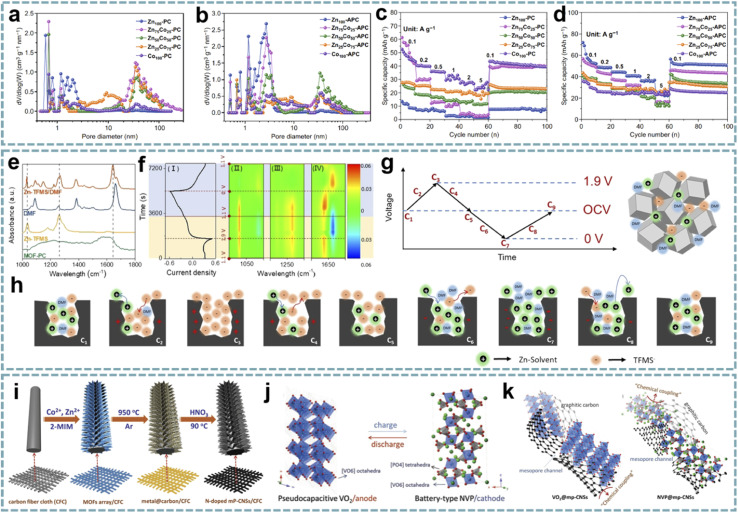
MOF-derived porous carbons for MIHCs. Pore size distribution curves of (a) Zn_*x*_Co_100−*x*_-PCs and (b) Zn_*x*_Co_100−*x*_-APCs. Rate capabilities at different current densities of (c) Zn_*x*_Co_100−*x*_-PCs and (d) Zn_*x*_Co_100−*x*_-APCs. Reproduced with permission from ref. 160. Copyright 2020, Springer. (e) FT-IR spectra of neat DMF, Zn-TFMS, MOF-PC, and Zn-TFMS/DMF electrolytes. (f) CV curve (I) at 0.5 mV s^−1^; 2D color-filled contour plots (II, III, and IV) from the *in situ* ATR-FTIR test of the ZIHC. (g, h) The reaction process schematic diagram of the adsorption/desorption of ions in the MOF-PC electrode in a Zn-TFMS/DMF electrolyte. Reproduced with permission from ref. 161. Copyright 2021, Wiley-VCH. (i) Schematic diagram of the fabrication of N-doped mesoporous graphitic carbon nanosheet arrays. (j) Schematic illustration of the NIC device using flexible VO_2_@mp-CNSs/CFC and NVP@mp-CNSs/CFC electrodes as the anode and cathode. (k) Schematic illustration of Na^+^ storage of VO_2_@mp-CNSs/CFC and NVP@mp-CNSs/CFC. Reproduced with permission from ref. 70. Copyright 2018, Wiley-VCH.

Ordered pore structures and hierarchical porosity are expected to be achieved through multiscale pore engineering of MOFs for the development and optimization of energy storage devices.^159^ Therefore, the customization and optimization of carbon pore size through MOF-derived porous carbon is a facile and efficient approach. Zou *et al.* performed directional control of the pore size and graphitization degree of ZIF-derived carbons by adjusting the molar ratio of Co/Zn ions in ZIFs,^160^ and explored the relationship between these properties and capacitive behavior (Fig. 7a–d). Interestingly, a suitable pore size of 1.5–3 nm can well match the size of solvated PF_6_^−^, and the strong adsorption/desorption interactions enhance the capacitive behavior of LiPF_6_. Meanwhile, the high degree of graphitization effectively improves the rate capability. Wang's group proposed a ZIHC based on an *N*,*N*-dimethylformamide (DMF) electrolyte containing Zn(CF_3_SO_3_)_2_,^161^ which consists of a MOF-derived porous carbon (MOF-PC) cathode and a Zn anode. By *in situ* attenuated total reflection-Fourier transform infrared (ATR-FTIR), the charge storage mechanism of the MOF-PC cathode was demonstrated, mainly involving the adsorption and exchange of anions/cations in macropores and this macropore-dominated charge storage enables fast charge/discharge even under high mass loading (Fig. 7e–h). Furthermore, based on the aprotic nature of DMF and the complex formed by Zn^2+^ and DMF in the electrolyte, Zn-corrosion can be avoided and dendrite-free Zn plating/stripping can be achieved. Therefore, this ZIHC has a long cycle life of over 9000 cycles and a Zn-utilization rate as high as 2.2%.

The coupling of suitable pore sizes and well-designed nanostructures exhibits surprising potential in MIHCs. 2D Co/Zn-MOF arrays were transformed into N-doped mesoporous carbon nanosheets (mp-CNS) (Fig. 7i).^70^ Based on this mesoporous carbon platform, VO_2_ and Na_3_V_2_(PO_4_)_3_ (NVP) were encapsulated in carbon shells, respectively (Fig. 7j). The uniform N-doping and high surface area in mp-CNS construct a 3D conductive network for fast electron/ion transport (Fig. 7k). A quasi-solid-state flexible SIHC based on the VO_2_@mp-CNS anode and NVP@mp-CNS cathode provides high energy/power density and an excellent cycle stability. Lu *et al.* fabricated N-doped carbon nanotube-coated porous carbon (CNT@PC) derived from a ZIF-67 precursor.^162^ Among them, porous carbon has a high specific surface area and ion adsorption sites, CNTs provide a conductive network, and the introduced N atoms add additional pseudocapacitance through surface redox reactions. In carbon structures, micropores, mesopores, and macropores coexist, and high mesopore volume provides ion transport channels and accommodates volume changes during ion insertion/extraction. Benefiting from these advantages, the CNT@PC-based SIHC and PIHC show great potential.

Based on the controllable structure/morphology of MOFs, MOF-derived onion-like carbons were developed.^87^ Remarkable structural properties, including high porosity, large surface area, high defect concentration, and accessibility of internal voids, ultimately lead to superior charge storage and Li^+^ transport capabilities in LIHCs. Porous carbon with a high pore volume and specific surface area was obtained by carbonization and acid washing of Zn-MOF assembled from 2,3,5,6-tetrafluoroterephthalic acid (H_2_TFBDC) and Zn^2+^.^163^ The microporous structure of porous carbon and PF_6_^−^ in the electrolyte can be well matched to obtain high capacitance. Additionally, *in situ* doping of O atoms provides more active sites. The LIHC device obtained by matching the porous carbon as a cathode with a sucrose hard carbon anode can deliver a high energy density of 157 W h kg^−1^ and a high power density of 40 kW kg^−1^ with an 85% capacity retention rate after 4000 cycles.

Well-designed pore sizes not only provide shortened paths for rapid transport of electrolyte ions, but also show large surface areas to facilitate the accessibility of active sites. The hierarchical pore structure based on MOF pore engineering can be tailored to meet the needs of ion transfer and storage. However, active sites cannot be created by the design of pores. The heteroatom doping that we introduce next will alleviate this deficiency.

#### 5.2.2. Role of heteroatom doping

The surface chemical state also plays a key role in the electrochemical performance of MIHCs. In addition to their tunable porosity, MOF-derived carbons are easily functionalized, especially by heteroatom doping, which provides abundant active sites for the storage of metal ions.^164–167^ Meanwhile, heteroatom doping can enhance electrical conductivity by tuning the energy band structure of carbons and modulating the chemical properties of the carbon material surface.^168^ Pairing MOF-derived heteroatom-doped carbon with battery-type electrodes is promising to obtain MIHC devices with simultaneous high energy/power density. In an early report, MOF-derived N-doped porous carbon as the cathode and a WO_3_/C microsphere anode constituted a high-performance LIHC.^69^ Another report used carbonized ZIF-8 as a cathode and assembled it with a nano-network structured Ni/NiO/C anode for robust LIHCs.^169^ Unfortunately, these reports do not provide insight into the role of heteroatom doping.

**Fig. 8:**
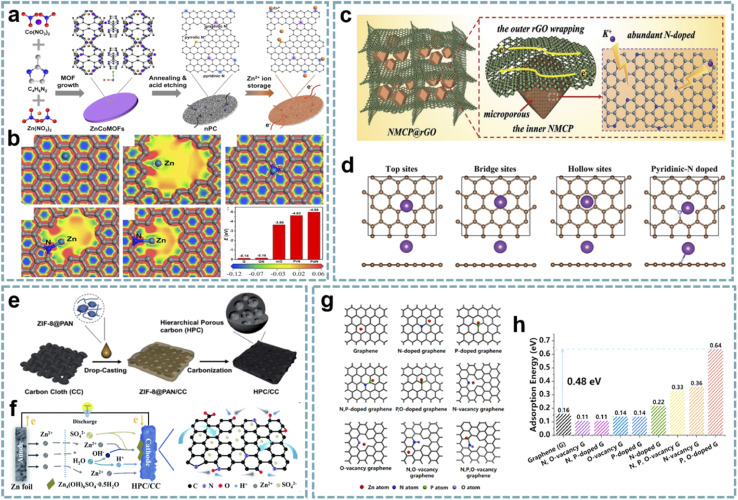
MOF-derived heteroatom-doped carbons for MIHCs. (a) Schematic of the fabrication of the nPC cathode for Zn^2+^ storage. (b) The electron density distributions of graphene, holey graphene with a micropore, graphitic N doped graphene, pyrrolic N doped graphene, and pyridinic N doped graphene, and the comparison of binding energies between Zn^2+^ and various models. Reproduced with permission from ref. 79. Copyright 2020, Elsevier. (c) Schematic configuration of NMCP@rGO. (d) Four calculation models after K^+^ absorption. Reproduced with permission from ref. 170. Copyright 2020, Wiley-VCH. (e) Schematic diagram of the preparation of HPC/CC. (f) Schematic diagram of the working mechanism of the HPC/CC-based aqueous ZIHC. Reproduced with permission from ref. 174. Copyright 2020, Royal Society of Chemistry. (g) DFT calculations unveiling the Zn atom adsorption capability for graphene models with various doping species. (h) Zn adsorption energy on graphene models. Reproduced with permission from ref. 89. Copyright 2022, Elsevier.

In recent years, the study of MOF-derived heteroatom-doped carbons has attracted great interest. Li and co-workers fabricated pyridinic N-rich porous carbons *via* pyrolysis and acid etching of ZnCo bimetallic MOFs (Fig. 8a).^79^ The micro/mesopores in porous carbon are beneficial for fast Zn^2+^ transport and storage, and the abundant N doping, especially pyridinic N with high binding energy to Zn^2+^, provides additional sites for Zn^2+^ storage (Fig. 8b). Additionally, the *in situ* formed CNTs and residual metal species enhance the electrical conductivity to facilitate fast electron transfer. The aqueous ZIHC based on this pyridinic N-rich porous carbon exhibits satisfactory zinc storage properties, including a high capacity of 302 mA h g^−1^ at 1 A g^−1^ and a long cycle life. In addition, the assembled quasi-solid-state ZIHC devices also exhibit excellent electrochemical performance and flexibility.

Integrating heteroatom doping with optimized carbon structures into one system will provide an excellent platform for the construction of advanced MIHCs. In Ruan's report,^170^ they constructed N-doped hierarchical dual-carbon structures, including a combination of 3D N-doped microporous carbon and 2D reduced graphene oxide (rGO) (Fig. 8c). The outer rGO improves the conductivity while maintaining the structural stability, and the optimal ratio of N doping in the inner microporous carbon provides more sites for K^+^ storage and induces pseudocapacitive behavior (Fig. 8d). Furthermore, the porous structure dominated by micropores can effectively buffer the volume change of the carbon framework during the K^+^ intercalation/deintercalation process. Benefiting from this synergistic effect, the PIHC based on this dual-carbon anode and AC cathode provide ultra-high energy/power density and high cycling stability (over 12 000 cycles) at a current density of 2 A g^−1^.

Considering that metal oxides can be used as sacrificial templates for the synthesis of MOF structures, Yuksel *et al.* initiated the formation of Zn^2+^ by etching the ZnO template with 2-methylimidazole, and then Zn^2+^ and ligand groups formed ZIF-8 on the surface of the ZnO template.^171^ After pyrolysis, necklace-like N-doped tubular carbons with high aspect ratios were synthesized. The high N content, large surface area, and high micropore volume of this structure provide an opportunity to fabricate high-performance aqueous ZIHCs, exhibiting an energy density of 189.6 W h kg^−1^ and a high specific capacitance of 341.2 F g^−1^ at 0.1 A g^−1^.

To combine the advantages of the pore structure of surface chemistry, Zhu *et al.* transformed the hybrid structure of ZIF-8, polypyrrole (PPy), and polyvinylpyrrolidone (PVP) into a 3D N-doped carbon nanocage based on a strategy of pyrolysis and activation.^172^ This approach achieves a high N doping content, hierarchical porosity, and open channels. Due to the advantages of the composition and structure, the ZIHC based on this carbon cathode shows enhanced Zn^2+^ storage capacity, excellent stability and a high energy/power density of 107.3 W h kg^−1^/16 647.7 W kg^−1^.

Single heteroatom doping provides improved conductivity and active sites. However, for higher electrochemical performance, dual-heteroatom doping may be more attractive, which is attributed to the synergistic effect between them. Xu's group proposed a solid-state gas-steamed approach for the conversion of ZIF-8 into open-wall carbon cages enriched with N and P doping.^83^ PH_3_ decomposed by NaH_2_PO_2_ caused defects and introduced phosphorus sources during the pyrolysis of ZIF-8. The open carbon cages offer great potential for cathodes for aqueous ZIHCs, where the open-walled structure provides channels for enhanced mass transport, and the active sites generated by N and P doping facilitate the chemisorption of Zn^2+^. As a validation, this carbon cage shows a wide operating voltage of 2.0 V and a high capacity of 225 mA h g^−1^ at 0.1 A g^−1^. Meanwhile, it exhibits a high capacity retention rate of 96.5% after 300 000 cycles (50 A g^−1^). The assembled soft-pack ZIHC device delivers a high energy density of 97 W h kg^−1^ and a high power density of 6.5 kW kg^−1^, and works well over a wide temperature range. Besides, Lei *et al.* proposed ZIF-8-derived N, O-doped porous carbon as a cathode for ZIHCs.^173^ Compared with N-doped carbon obtained by direct carbonization of ZIF-8 in most reports, they added KOH for activation after carbonization, followed by secondary annealing, and obtained N, O dual-doped carbon after soaking in HNO_3_. It provides excellent zinc storage capacity due to its high surface area, hierarchical porosity, and abundant active sites. In another report,^174^ N, O dual-doped hierarchical porous carbon integrated with carbon cloth was developed. The synthesis process involves the combination of ZIF-8/polyacrylonitrile (PAN) with carbon cloth, and a carbonization step (Fig. 8e). Benefitting from its suitable pore size distribution, high specific surface area, interconnected conductive network and N, O heteroatom doping, this hierarchical porous carbon structure exhibits fast electron/ion transport as a cathode for ZIHCs and provides high capacity (138.5 mA h g^−1^ at 0.5 A g^−1^), excellent rate capability (75 mA h g^−1^ at 20 A g^−1^), and robust stability (over 10 000 cycles). More importantly, the mechanism of reversible chemical absorption/desorption of dual cations (Zn^2+^ and H^+^) on the cathode to increase capacity during charge/discharge is revealed (Fig. 8f).

Multi-heteroatom doping is expected to provide more exciting electrochemical performance due to the complementary advantages and enhanced synergy between multiple heteroatoms. Leng *et al.* coated ZIF-8 with phosphatidylcholine and prepared N, P, O tri-doped carbon nanocage cathodes for ZIHCs after carbonization.^89^ Density functional theory (DFT) calculations show that the most stable adsorption sites for Zn atoms are N vacancies and P–O sites (Fig. 8g and h). Due to the high surface area, hierarchical porosity, and multi-heteroatom doping of the cathode, the ZIHC provides a high specific capacitance of 310 F g^−1^ at 0.5 A g^−1^.

In order to fabricate carbon materials with higher electrochemical activity, a leap from single-heteroatom doping to multi-heteroatom doping has been achieved. Different heteroatom doping yields various heteroatom groups, and their degree of modulation of the carbon structure is significantly different, which leads to a rational combination between them to produce enhanced synergistic effects. It is important to note that as the number of heteroatoms increases, their respective roles become more difficult to understand.

#### 5.2.3. Role of oxygen functional groups

Heteroatom doping is generally regarded as an effective strategy to improve the electrochemical performance of carbon materials. However, the effect of oxygen functional groups is often overlooked. Oxygen functional groups can provide opportunities to further improve the electrochemical performance, and abundant oxygen functional groups can provide additional capacitive adsorption sites for metal ions, which can significantly increase the capacity.^175–178^ Lu's research group proposed that the C–O bond participates in the Zn storage process by forming C–O–Zn with Zn^2+^, while the additional N doping can significantly promote the chemisorption of Zn^2+^.^179^ Shao *et al.* demonstrated that carboxyl and carbonyl groups can significantly improve Zn^2+^ chemisorption, pseudocapacitive redox activity, and aqueous electrolyte wettability.^180^ It has also been reported that the hydroxyl group promotes the storage behavior of Zn^2+^ in porous carbon, and the pseudocapacitive contribution and the chemisorption of Zn^2+^ have been investigated.^181^ Yan's group demonstrated that oxygen-containing functional groups in carbon nanosheets provide defects and additional active sites. These features facilitate K^+^ storage and enable high-performance PIHCs.^182^

**Fig. 9:**
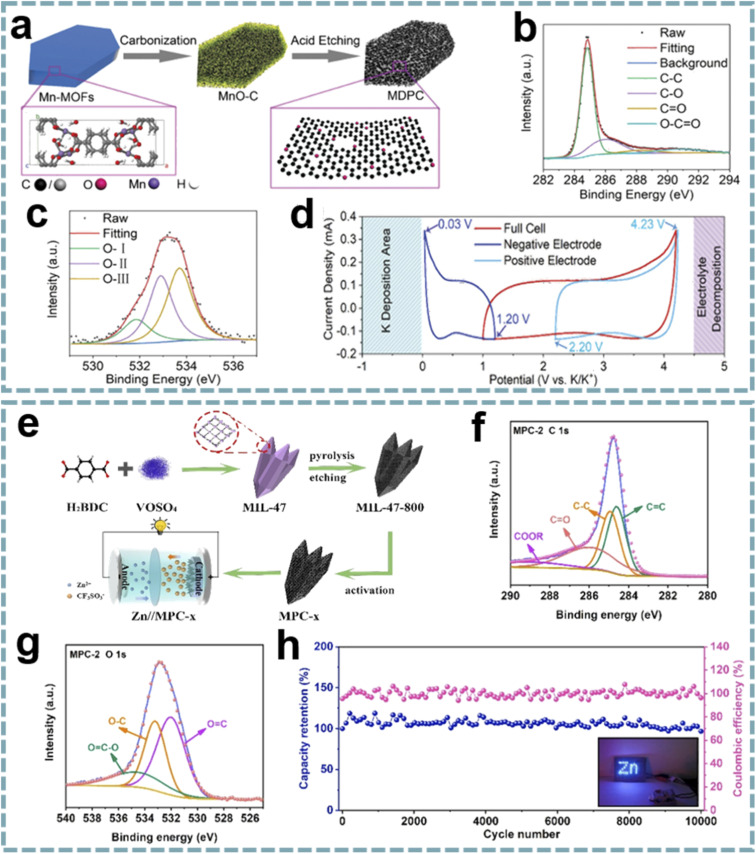
MOF-derived oxygen-functional carbons for MIHCs. (a) Schematic diagram of the synthesis of a MDPC. (b) C 1s and (c) O 1s XPS spectra of the MDPC. (d) CV profiles based on a three-electrode device at 2 mV s^−1^. Reproduced with permission from ref. 185. Copyright 2020, Wiley-VCH. (e) Synthesis schematic of MPC and construction of Zn//MPC-*x* ZIHC. High-resolution XPS spectra of (f) C 1s and (g) O 1s of MPC-2. (h) Cycling performance and coulombic efficiency at 10 A g^−1^ of the Zn//MPC-2 ZIHC device. Reproduced with permission from ref. 186. Copyright 2021, Elsevier.

Although some O-doped carbons for MIHCs are presented in the chapter on heteroatom doping, these reports do not provide insight into the role of oxygen functional groups. Given the important properties of oxygen functional groups,^183^ it is necessary to emphasize the unique position of oxygen functional groups in MIHCs. Due to the selectable ligands in MOFs, as well as the addition of guest molecules during carbonization, the functionalization of carbon surfaces, especially oxygen functional groups, can be easily achieved.^184^ In Shao's report, they designed a porous carbon microsheet with highly disordered and interlayer-expanded structure by carbonization and acid treatment of Mn-MOF (Fig. 9a).^185^ Oxygen doping was demonstrated by X-ray photoelectron spectroscopy (XPS), including C=O quinone groups, C–OH hydroxylic groups and –O–C=O carboxyl groups (Fig. 9b and c). The presence of oxygen functional groups can enhance electrolyte wettability to increase the active surface area and provide additional active sites for K^+^ storage (Fig. 9d). By using porous carbon microsheets as an anode and assembling a PIHC with an AC cathode, a high energy density of 120 W h kg^−1^, a maximum power density of 26 kW kg^−1^ and a long-term cycle life of over 120 000 cycles were provided. In another report, sharpened pencil-like hierarchical porous carbons were derived from the carbonization of MIL-47 (V), followed by acid etching and KOH activation in sequence (Fig. 9e).^186^ The oxygen functional groups on the carbon surface were analyzed by using XPS spectra (Fig. 9f and g), including O=C, O–C and O=C–O. Oxygen-containing functional groups can form a hydrophilic surface, thereby reducing the interface resistance and facilitating the diffusion of electrolyte ions. Meanwhile, the oxygen-containing functional groups of quinone type (C=O) can provide pseudocapacitance for carbon materials through structural conversion between benzoquinone and hydroquinone. The GCD curves exhibit a slightly distorted triangular shape, demonstrating the additional contribution of pseudocapacitance to charge storage. A ZIHC based on this carbon cathode provides a specific capacitance of 289.2 F g^−1^ at 0.2 A g^−1^ and can achieve a maximum energy density of 130.1 W h kg^−1^, a maximum power density of 7.8 kW kg^−1^ and good cycle stability (Fig. 9h). These outstanding properties are attributed to the high specific surface area, abundant oxygen-containing functional groups and hierarchical porous structure of the carbon cathode.

### 5.3. MOF-derived metal/carbons

Benefiting from the designable metal nodes and organic ligands of MOFs, diverse functional materials are formed from different MOFs after annealing at a high temperature in an inert atmosphere. Among them, the organic ligands are decomposed into porous carbon, and the existing heteroatoms are doped into the carbon skeleton. Metal nodes are usually reduced to metal nanoparticles. However, under the action of other atmospheres (*e.g.*, NH_3_ and PH_3_) or guest molecules, specific metal compounds are produced. Besides inheriting the porous structure and initial morphology of MOFs, the metal species obtained after annealing in an inert gas are usually uniformly dispersed in the carbon matrix. The porous carbon framework improves the overall conductivity of the material and avoids the dissolution and aggregation of nanoparticles during electrochemical reactions. This hybrid structure is significantly different from the traditional carbon coating on the surface of metal species, which greatly improves the electrochemical performance of metal species. These MOF-derived metal/carbons with different functionalities exhibit satisfactory performance in MIHCs.^187^

#### 5.3.1. MOF-derived metal nanoparticle/carbons

**Fig. 10:**
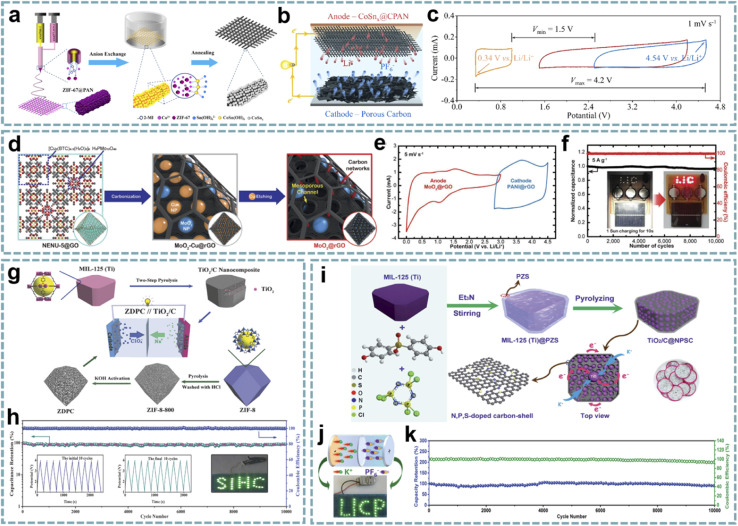
MOF-derived metal/carbons for MIHCs. (a) Schematic diagram of the fabrication process of CoSn_*x*_@CPAN nanofibers. (b) Schematic of the configuration of a full-cell LIHC. (c) CV profiles from a three-electrode device. Reproduced with permission from ref. 189. Copyright 2020, Elsevier. (d) Schematic diagram of the fabrication process of the MoO_2_@rGO anode. (e) CV curves of the MoO_2_@rGO anode and PANI@rGO cathode at a scan rate of 5 mV s^−1^. (f) Charge–discharge cycling stability at 5 A g^−1^ with a wireless charging kit charged by a solar cell. Reproduced with permission from ref. 78. Copyright 2020, Wiley-VCH. (g) Construction process of the TiO_2_/C anode and ZDPC cathode. (h) Cycling performance and coulombic efficiency of the TiO_2_/C//ZDPC SIHC under 1 A g^−1^. Reproduced with permission from ref. 193. Copyright 2018, Wiley-VCH. (i) Schematic diagram of the preparation process of TiO_2_/C@NPSC. (j) LED arrays powered by the PIHC device. (k) Cycling performance and coulombic efficiency. Reproduced with permission from ref. 80. Copyright 2020, Royal Society of Chemistry.

Although MOFs provide a superior platform for fabricating carbon composites, the synthesis of homogeneously dispersed metal nanoparticles of suitable size on a carbon matrix requires a suitable strategy. In Jia's report, N-doped carbon-coated Co nanoparticles (Co@N–C) were prepared by carbonization of ZIF-67.^188^ Among them, Co nanoparticles promoted the *in situ* formation of carbon nanotubes on the surface of carbon polyhedrons. Due to the multilayer structure and ideal composition, Co@N–C offers superior electron/ion transport capability and Li^+^ trapping capability and shows a high pseudocapacitive contribution. The assembled LIHC achieves an excellent energy density of 125.28 W h kg^−1^ and a power density of 10 kW kg^−1^. Bian *et al.* developed a general anion exchange strategy for the construction of MOF-derived nanostructures within polymer fibers (Fig. 10a).^189^*In situ* conversion results in a homogeneous dispersion of the active components. Among them, the hybrid structure of CoSn_*x*_ nanoparticles with an interconnected carbon framework was used as the anode for LIHCs, showing high capacity and fast electron/ion diffusion, and delivering a high energy density of 143 W h kg^−1^ (Fig. 10b and c).

#### 5.3.2. MOF-derived metal oxide/carbons

Metal oxide/carbon formed by the *in situ* transformation of MOFs not only achieves a uniform distribution of metal oxides in the carbon framework, but also achieves overall high electrical conductivity and desirable functionality. Dubal *et al.* fabricated a hybrid structure of MnO_2_ embedded in graphene-like carbon nanosheets (MnO_2_@C-NS) based on Mn-based MOFs.^74^ Among them, the hierarchical porous carbon nanosheets buffer the volume change of MnO_2_ and prevent its aggregation while providing high electrical conductivity. A LIHC based on the MnO_2_@C-NS anode and an ultrathin porous carbon nanosheet cathode delivers a high energy density of 166 W h kg^−1^ at 550 W kg^−1^. Low-boiling metals act as nodes of MOFs, and the volatilization after high-temperature annealing is favorable for the formation of a porous carbon matrix. Therefore, bimetallic ZnMn-MOF was used for *in situ* conversion to MnO-encapsulated porous carbon (MnO/PC).^190^ The activation of the Zn metal centers endows the carbon matrix with a highly porous structure. Porous carbon provides sufficient voids to buffer the volume change of MnO, thereby preventing the structural damage of MnO during cycling and also improving the overall conductivity. MnO/PC exhibits extraordinary lithium storage ability. More importantly, the MnO/PC anode can be well-matched with AC, and the LIHC exhibits a high energy density of 153.6 W h kg^−1^ at 210 W kg^−1^ and maintains 71.8 W h kg^−1^ at 63 kW kg^−1^. Kang's group designed a MoO_2_@rGO mesoporous structure from NENU-5 (NENU stands for Northeast Normal University) (Fig. 10d).^78^ The mesoporous channel facilitates the permeation of Li^+^ between the electrolyte and MoO_2_, where MoO_2_ is embedded in the conductive carbon network to provide a fast electron/ion transport pathway. In addition, polyaniline (PANI) was grown on rGO and served as the cathode. The MoO_2_@rGO//PANI@rGO LHIC achieves an ultra-high energy density (242 W h kg^−1^), high power density (28 750 W kg^−1^), and long-term stability over 10 000 cycles (Fig. 10e and f). Jiao *et al.* obtained M-Nb_2_O_5_@C/rGO by pyrolyzing the composite of Nb-MOF and GO.^191^ The porous structure enables rapid diffusion of Li^+^ during charging and discharging. rGO acts as both a buffer layer for volume expansion and a conductive network. The M-Nb_2_O_5_@C/rGO//AC LIHC provides a maximum energy density of 71.5 W h kg^−1^, high power density and good cycling stability. Cheng and co-workers demonstrated an LIHC integrated with a carbonized nickel cobalt oxide (cNiCo_2_O_4_) anode and vertically aligned carbon nanoflake (VACNF) cathode.^192^ MOF-derived bimetallic oxide provides fast conversion reactions and high pseudocapacitance, while the carbon cathode has a high electrochemically active area. The cNiCo_2_O_4_//VACNF device delivers a high energy density of 136.9 W h kg^−1^ at a high power density of 40 kW kg^−1^.

Coupling advanced cathode and anode materials constructed from MOFs provides an ideal electrode combination for high-performance MIHCs. Yan's group demonstrated a SIHC consisting of a TiO_2_/C anode and a 3D porous carbon cathode,^193^ which was designed from MIL-125 (Ti) and ZIF-8, respectively (Fig. 10g). The robust structure of TiO_2_/C leads to fast kinetics and excellent stability. The 3D porous carbon inherits the hierarchical porous structure and high surface area of ZIF-8, showing good capacitive performance. Therefore, the SIHC achieves a maximum energy density of 142.7 W h kg^−1^ and a maximum power density of 25 kW kg^−1^. Meanwhile, it has a capacity retention rate of more than 90% after 10 000 cycles (Fig. 10h). In fact, employing two different MOF precursors adds complexity to the experiments. The cathode and anode derived from a single MOF precursor appear to be more feasible. Bao's group proposed a “two-for-one” strategy to fabricate a TiO_*x*_N_*y*_/C anode and N-doped hierarchical porous carbon (NHPC) cathode derived from NH_2_-MIL-125 (Ti).^72^ The small size and pseudocapacitive properties of TiO_*x*_N_*y*_ enable it to exhibit superior sodium storage performance, and the NHPC has a highly porous structure, high specific area, and high N doping content. By coupling TiO_*x*_N_*y*_/C and the NHPC, the assembled SIHC provides excellent performance.

When metal oxides are encapsulated in a heteroatom-doped carbon matrix, besides maintaining the inherent advantages of metal oxide/carbon, the abundant functional groups provided by heteroatom doping provide additional active sites for ion storage and achieve better electrical conductivity and wettability. Bu's group reported a V_2_O_3_/N-doped porous carbon nanorod anode derived from V-MOF and used for SIHCs.^73^ The small-sized V_2_O_3_ particles and graphitic-N-rich carbon matrix show excellent sodium storage properties. The device with an AC cathode provides excellent cycling stability and exhibits a high energy density of 38.7 W h kg^−1^ at 5805 W kg^−1^. Li *et al.* fabricated TiO_2_/C confined in N, P, S co-doped carbon by pyrolyzing polymer-modified MIL-125 (Ti) (Fig. 10i).^80^ The unique dual-carbon heterostructure effectively buffers the volume change of TiO_2_ during charging and discharging and provides abundant active sites, thereby enhancing the pseudocapacitive-dominated K^+^ storage. When integrated with a ZIF-8-derived carbon cathode, the assembled PIHC provides an energy density of 114 W h kg^−1^ and a power density of 21 kW kg^−1^. In addition, it also exhibits cycle stability over 10 000 cycles (Fig. 10j and k).

#### 5.3.3. MOF-derived metal sulfide/carbons

**Fig. 11:**
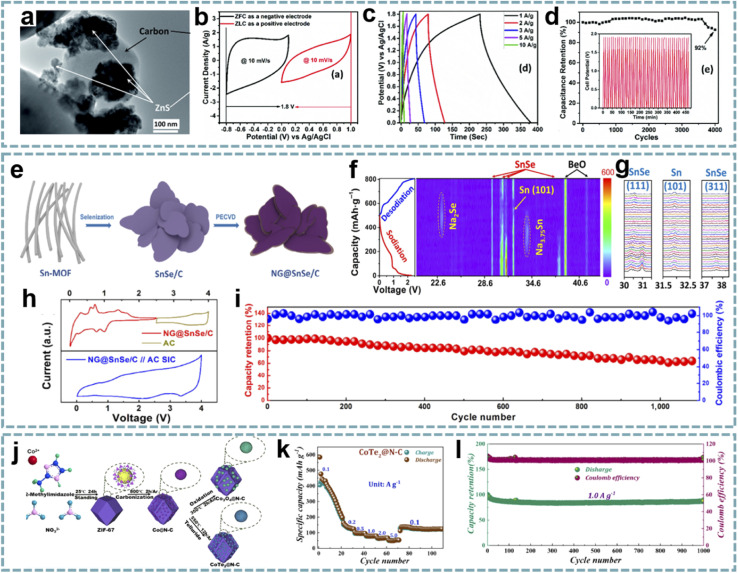
MOF-derived metal/carbons for MIHCs. (a) TEM image of ZnS@C. (b) CV profiles from a three-electrode device. (c) GCD curves of the SIHC at different current densities. (d) Cycling stability of the SIHC for 4000 cycles at 20 A g^−1^. Reproduced with permission from ref. 194. Copyright 2021, Royal Society of Chemistry. (e) Schematic diagram of the preparation process of NG@SnSe/C. (f) *Operando* XRD patterns of NG@SnSe/C operated for a full cycle of charge/discharge. (g) Corresponding XRD patterns within a selected 2*θ* range. (h) CV profiles of NG@SnSe/C and AC in half cells and a full cell of SIHCs. (i) Cycling performance of the SIHC at 0.5 A g^−1^. Reproduced with permission from ref. 77. Copyright 2019, Springer. (j) Schematic diagram of the synthesis process of CoTe_2_@N–C. (k) Rate performance of CoTe_2_@N–C. (l) Long cycling performance and coulombic efficiency of the LIHC. Reproduced with permission from ref. 197. Copyright 2021, Elsevier.

Metal sulfides generally store charge through redox reactions. However, metal sulfides have poor electrical conductivity, which are prone to structural change during charge and discharge. Porous carbon coating is a solution to obtain high conductivity and stability. More importantly, this structure facilitates faster charge transport and volume change control during charge and discharge. The natural advantages of MOFs provide opportunities for designing metal sulfide/carbon structures. Shrivastav *et al.* designed ZnS@C and porous carbon based on ZIF-8, and constructed a SIHC.^194^ The *in situ* transformation of the MOF achieves a uniform distribution of ZnS in the porous carbon matrix (Fig. 11a). The reasonable match of these two materials leads to superior performance in SIHCs (Fig. 11b–d).

#### 5.3.4. MOF-derived metal selenide/carbons

Compared with metal oxides and metal sulfides, metal selenides show better electrical conductivity and weaker M–Se bond strength, which endow them with superior kinetic properties. Lu and co-workers designed MOF-derived SnSe/C and encapsulated in N-doped graphene (NG) by chemical vapor deposition (CVD) (Fig. 11e).^77^ The inner carbon framework and outer NG carbon shell provide NG@SnSe/C with excellent structural stability and electrical conductivity. N doping effectively improves the adsorption/diffusion of Na^+^. The sodium storage mechanism of SnSe was deeply investigated by *in situ* X-ray diffraction (XRD) (Fig. 11f and g), and the NG@SnSe/C//AC SIHC achieved a high energy density of 115.5 W h kg^−1^ and a power density of 5742 W kg^−1^, as well as good cycling performance (Fig. 11h and i).

Advanced structural and compositional designs in electrode materials are critical for achieving high performance. Chen *et al.* designed porous yolk–shell ZnSe@CoSe_2_ based on ZIF-8@ZIF-67.^195^ The space of the yolk–shell structure provides a buffer for the volume change generated by the insertion of Li^+^, and the N-doped carbon matrix increases the lithium storage sites and shortens the ion diffusion path. Therefore, the LIHC integrated with AC shows high energy/power density and excellent cycling performance. Another report demonstrates the functionality of multi-heteroatom doping.^196^ Multi-heteroatom-doped Cu_1.8_Se/C was synthesized by selenization of Cu-MOF. The enhanced sodium storage performance is attributed to its high specific surface area, N, P, O co-doping, and uniform distribution of Cu_1.8_Se. The Cu_1.8_Se/C//AC SIHC device provides a high energy density of 65.8 W h kg^−1^ at 81.4 kW kg^−1^, and a long-term stability over 3000 cycles (2 A g^−1^).

#### 5.3.5. MOF-derived metal telluride/carbons

Metal tellurides have high theoretical capacities, but they also face a series of problems, such as electrode structure destruction caused by volume change during the charge–discharge process, limited electron/ion transport capacity, and formation of unstable solid electrolyte interface (SEI) films in electrolytes. Similar to other metal chalcogenides, the design of porous carbon-coated metal tellurides through MOFs can effectively address the above problems. In Zhang's work, ultrafine CoTe_2_ encapsulated in N-doped porous carbon was synthesized after sequential carbonization and tellurization of ZIF-67 (Fig. 11j).^197^ The *in situ* encapsulation achieves agglomeration-free CoTe_2_, and the N-doped carbon framework enhances the structural stability of CoTe_2_ and overall electrical conductivity, resulting in superior lithium storage performance (Fig. 11k). When assembled with a hierarchical porous carbon cathode into a LIHC, a maximum energy density of 144.5 W h kg^−1^ was achieved along with excellent power density and long cycle life (Fig. 11l).

#### 5.3.6. MOF-derived metal nitride/carbons

**Fig. 12:**
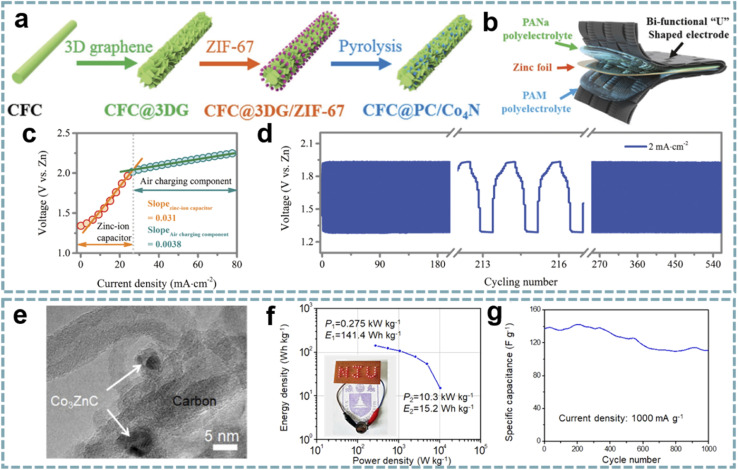
MOF-derived metal/carbons for MIHCs. (a) Schematic diagram of the preparation of CFC@PC/Co_4_N. (b) The designed architecture of an “air chargeable” ZIHC. (c) Galvanodynamic charge curves and (d) GCD cycling stability of the integrated system. Reproduced with permission from ref. 199. Copyright 2019, Wiley-VCH. (e) TEM image of Co_3_ZnC@NC. (f) Ragone plot of the LIHC. (g) Cycling performance of the LIHC at 1 A g^−1^. Reproduced with permission from ref. 71. Copyright 2018, Elsevier.

Metal nitrides have high electrical conductivity similar to metals and show great promise in EES and energy conversion.^198^ Zhi's group proposed an “air chargeable” ZIHC system.^199^ Among them, flexible bifunctional (energy conversion and storage) electrodes were obtained by depositing 3D graphene on carbon fiber cloth and growing ZIF-67, followed by final pyrolysis (Fig. 12a). In the product, carbon polyhedra are wrapped with interconnected carbon networks. The derivatives of ZIF-67 are rich in Co_4_N, and this Co_4_N embedded in the porous carbon matrix provides excellent electrochemical activity. Meanwhile, this material exhibits bifunctional properties as a ZIHC cathode for energy storage and an air electrode for energy conversion. The “air chargeable” energy storage system shows low dependence on the environment and great potential as a self-charging energy storage device (Fig. 12b–d).

#### 5.3.7. MOF-derived metal carbide/carbons

Metal carbides are candidates for electrode materials due to their good electrical conductivity, excellent chemical stability, and fast kinetics.^200^ To bridge the kinetic gap between the anode and cathode, Zhu *et al.* fabricated Co_3_ZnC-encapsulated mesoporous N-doped carbon (Co_3_ZnC@NC) by direct pyrolysis of ZnCo-ZIF and used as the anode for a LIHC (Fig. 12e).^71^ The superb lithium storage performance of Co_3_ZnC@NC is attributed to the pseudocapacitive properties as well as the electron/ion transport channels created by the hierarchical porous structure. Biomass-derived heteroatom-doped microporous carbon (MPC) was used as the cathode. The good matching of the anode and cathode alleviates the kinetic imbalance. Therefore, the Co_3_ZnC@NC//MPC LIHC provides a high energy density of 141.4 W h kg^−1^ and a high power density of 10.3 kW kg^−1^ over a wide operating voltage range (1.0–4.5 V), as well as long cycle life (Fig. 12f and g).

### 5.4. Summary

The fabrication of MOF derivatives requires *in situ* transformation of MOFs, involving chemical treatments and thermal treatments. Among them, heat treatment has received more extensive attention. The metal nodes of MOFs are transformed into diverse metal species at high temperatures, and they are uniformly distributed in the products. The transformation products of organic ligands are influenced by the pyrolysis atmosphere. Inert gases are usually chosen as the pyrolysis atmosphere, which leads to the transformation of organic ligands into porous carbons. Meanwhile, the heteroatoms in the ligands are doped into porous carbons after the carbonization of the MOFs, resulting in richer microstructures and better electrochemical performance. The performance of MIHCs largely depends on the electrode materials, and the structure of the electrode material is a key factor determining its performance. In addition to electrochemical reactions at the electrode surface, some of them also involve bulk transport. In addition, electrode materials need to reversibly store more metal ions. Therefore, electrode materials need to be able to adsorb and release metal ions rapidly and reversibly, depending on the ion transport channel. To overcome these challenges, designs of novel nanostructures are proposed, including 0D, 1D, 2D and 3D architectures. There are obvious differences between them. For example, they provide different electron/ion transport paths along the structure, resulting in different electron/ion conductivities. The 0D architectures can relieve the mechanical stress due to the volume change of the active materials. The 1D architectures are able to provide efficient electron transport paths along the length, enabling fast charge transport. Furthermore, they are able to limit the mechanical strain induced by volume change to a low level in the radial direction. The large specific surface area of 2D architectures is beneficial to fully expose the active species and increase the contact with the electrolyte, and provide excellent structural stability. 3D architectures usually consist of 0D, 1D or 2D architectures. Compared with the other three architectures, the 3D architectures can provide efficient conductive networks for active materials, leading to improved electrochemical performance. MOF derivatives as electrode materials for MIHCs are summarized in Table 3.

**Table 3:** MOF derivatives as electrode materials for MIHCs

MOF precursors	MOF derivatives	Applications	Configurations	Energy density (W h kg^−1^)	Power density (W kg^−1^)	Cycling stability	Ref
**MOF-derived metal compounds**							
Fe-MOF	α-Fe_2_O_3_ HPNP	LIHC	α-Fe_2_O_3_ HPNP//carbon spheres	107	9680	2500 cycles (1 A g^−1^)	149
CoZn-ZIF	Ti_3_C_2_@Co_3_O_4_/ZnO	LIHC	Ti_3_C_2_@Co_3_O_4_/ZnO//AC	196.8	3500	6000 cycles (2 A g^−1^)	86

**MOF-derived carbons**							
Fe-BTC	Onion-like carbons	LIHC	Onion-like carbon//AC	224	14 436	4000 cycles (1 A g^−1^)	87
ZIF-8	N-doped microporous carbon@rGO	PIHC	N-doped microporous carbon@rGO//AC	158.1	19 091	12 000 cycles (2 A g^−1^)	170
ZIF-8	Necklace-like N-doped carbon	ZIHC	Zn//necklace-like N-doped carbon	189.6	11 261.3	50 000 cycles (5 A g^−1^)	171
ZIF-8	N, P-doped open carbon cage	ZIHC	Zn//N, P-doped open carbon cage	97	6500	300 000 cycles (50 A g^−1^)	83
ZnCo-MOF	N-doped porous carbon	ZIHC	Zn//N-doped porous carbon	157.6	—	10 000 cycles (5 A g^−1^)	79
ZIF-8	N, O-doped carbon	ZIHC	Zn//N, O-doped carbon	110	20 000	10 000 cycles (5 A g^−1^)	174
ZIF-8	N, P, O tri-doped carbon	ZIHC	Zn/carbon cloth@ZIF-8//N, P, O tri-doped carbon	43	137 900	10 000 cycles (10 A g^−1^)	89
Mn-MOF	Porous carbon	PIHC	Porous carbon//AC	120	26 000	120 000 cycles (2 A g^−1^)	185

**MOF-derived metal/carbons**							
ZIF-67	CoSn_*x*_/carbon nanofibers	LIHC	CoSn_*x*_/carbon nanofiber//porous carbon	143	22 800	5000 cycles (2 A g^−1^)	189
NENU-5	MoO_2_@rGO	LIHC	MoO_2_@rGO//PANI@rGO	242	28 750	10 000 cycles (5 A g^−1^)	78
Mn-MOF	MnO_2_@C	LIHC	MnO_2_@C//nanoporous carbon	166	3500	5000 cycles (1 A g^−1^)	74
Nb-MOF	Nb_2_O_5_@C/rGO	LIHC	Nb_2_O_5_@C/rGO//AC	71.5	3900	2500 cycles (0.2 A g^−1^)	191
NH2-MIL-125 (Ti)	TiO_*x*_N_*y*_/C	SIHC	TiO_*x*_N_*y*_/C/N-doped carbon	80	4000	1200 cycles (0.8 A g^−1^)	72
MIL-125 (Ti)	TiO_2_/C@NPSC	PIHC	TiO_2_/C@NPSC//AC	114	21 000	10 000 cycles (1 A g^−1^)	80
V-MOF	V_2_O_3_/N-doped carbon	SIHC	V_2_O_3_/N-doped carbon//AC	63	5805	5000 cycles (1 A g^−1^)	73
ZIF-8	ZnS@C	SIHC	Nanoporous carbon//ZnS@C	38.3	—	4000 cycles (20 A g^−1^)	194
Sn-MOF	NG@SnSe/C	SIHC	NG@SnSe/C//AC	115.5	5742	1100 cycles (0.5 A g^−1^)	77
ZIF-67	CoTe_2_@N–C	LIHC	CoTe_2_@N–C//porous carbon	144.5	10 000	1000 cycles (1 A g^−1^)	197
ZnCo-ZIF	Co_3_ZnC@NC	LIHC	Co_3_ZnC@NC//microporous carbon	141.4	10 300	1000 cycles (1 A g^−1^)	71

## 6. Optimization strategies for electrode materials

**Fig. 13:**
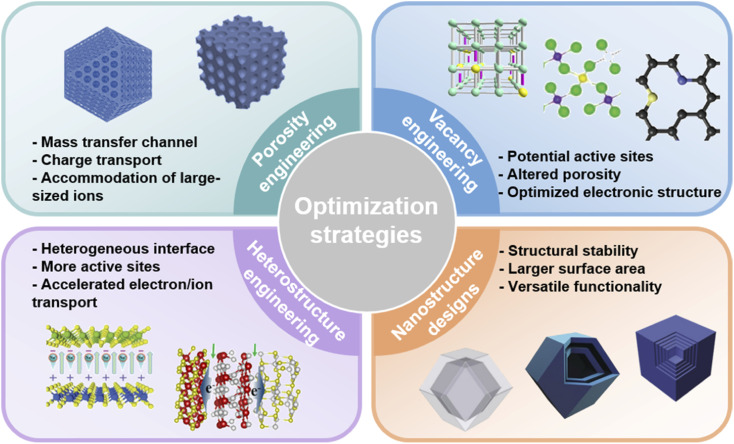
Schematic diagram of the optimization strategies for electrode materials.

MIHCs are considered as a competitive EES system. They couple battery-type electrodes and capacitor-type electrodes to provide battery-like high capacities and capacitor-like fast charge/discharge rates. The key to constructing high-performance MIHCs is to pair appropriate anode and cathode materials to achieve well-matched capacity and kinetic behavior. With this in mind, it is crucial to develop advanced anode and cathode materials to improve the rate capability of battery-type electrodes and the capacitance of capacitive-type electrodes. Fortunately, MOFs provide a multifunctional platform for the rational construction of MIHCs. Despite great progress, especially as electrode materials, MOFs and their derivatives still suffer from inherent shortcomings; typically, the pore structure, conductivity, and functionality of pristine MOFs are unsatisfactory. Meanwhile, the intrinsic properties of a microporous-dominant structure, irreversible aggregation of nanoparticles, and poorly controlled structural evolution exist in MOF derivatives. These factors largely hinder their electrochemical performance. To overcome these limitations, structural design and component optimization can greatly alleviate the above problems. Here, the focus is on component–structure–property interaction relationships. These optimization strategies include porosity engineering, vacancy engineering, heterostructure engineering, and nanostructure designs (Fig. 13).

### 6.1. Main challenges

For MIHCs, the matching of battery-type materials and capacitor-type materials is an important factor for obtaining high energy/power densities. Therefore, there is a need to develop advanced electrode materials that combine high energy/power, long-cycle stability, and a low self-discharge rate. Meanwhile, the balance of the capacity/kinetics of the cathode/anode can maximize the energy/power density of the device and maintain a long cycle life.

The slow kinetics of battery-type materials leads to enormous difficulties in realizing high-performance MIHCs. Therefore, accelerating the ion transport rate is the key to improving the kinetics of battery-type electrodes. For battery-type electrodes, the modification and design of composite materials are common methods to improve conductivity and speed up ion diffusion. However, these traditional strategies may increase the fabrication cost or sacrifice the energy density.

On the other hand, the capacity of capacitor-type materials needs to be optimized. Capacitor-type materials are usually based on carbon materials and pseudocapacitive materials. The lower specific capacitance of carbon materials largely limits the energy density of MIHCs. There is an urgent need to optimize the pore structure to ensure the compatibility between carbon materials and electrolyte ions. In addition, tuning of the microstructure, design of nanostructures and the aforementioned heteroatom doping are included. Besides the optimization of carbon materials, the development of pseudocapacitive materials with high specific capacitance is also an effective approach to improve the energy density of MIHCs, because pseudocapacitance is based on reversible surface faradaic reactions to store charges rather than ion adsorption/desorption. Unfortunately, the rate performance and cycling performance of pseudocapacitive materials are not as expected relative to carbon materials.

### 6.2. Advanced optimization strategies

#### 6.2.1. Porosity engineering

MOFs possess ordered micro/mesoporous structures and abundant chemical compositions. These properties enable highly designable pore structures (volume, pore size, *etc.*) at large scales (from sub-nanometers to tens of nanometers).^201^ The high specific surface area and interconnected/network porosity provide potential active sites as well as mass transfer channels. Large pore volumes and specific surface areas can be easily achieved by judicious design of metal nodes and organic linkers. Despite the poor conductivity of conventional MOFs, this issue is expected to be overcome by highly conjugated organic linkers.^202^ Therefore, the optimization of pore structures through multiscale pore engineering and reticular chemistry design endows them with great potential in MIHCs. Furthermore, annealing of MOFs in different atmospheres can produce diverse MOF derivatives, including porous carbon, metal compounds, metal compounds/carbon, *etc.*, which can inherit the structural properties of MOFs (*e.g.*, large specific surface area and high porosity).^203^ Well-designed MOF precursors and altered process parameters can control the structure and composition of MOF derivatives. The relatively large interior space can withstand significant volume changes of the active material.

MIHCs can be viewed as a combination of SCs and MIBs. In terms of SCs, a large specific surface area and high electrical conductivity are required to maximize the number of adsorbed ions. Meanwhile, appropriate pore size distribution is demanded for fast mass transfer and high volume-normalized power/energy density. The quantum confinement effects that emerge can be tuned to improve performance when the size of the pores drops to the nanoscale or even sub-nanometer scale.^204^ At the level of MIBs, it is crucial to improve rate performance through proper pore size distribution to facilitate charge/mass transfer. For instance, metal ions of larger sizes (*i.e.*, Na^+^ and K^+^) require larger average pore sizes in order to accommodate the corresponding ions for further operation.^159^

To regulate the functions and properties of MOFs, it is necessary to adjust the pore structure (*e.g.*, topology, size, and volume) and pore chemistry (*e.g.*, the composition or function of metal secondary building units and ligands). The pore structure has extraordinary implications for the function of MOFs. For example, micropores (<2 nm) can tune the confinement effect and thus enhance the performance of SCs, and mesopores (2–50 nm) feature a larger size than micropores, which is beneficial to facilitate mass transport within the network. The most straightforward way to obtain mesopores in MOFs is the design of ligands, thereby enlarging the original micropores by lengthening the ligands for retrosynthetic design or *de novo* reticular design by modulating topologies.^201^ Macropores (>50 nm) can facilitate mass transfer and thus be beneficial for rate performance.^205^

Pristine MOFs are chemically/heat-treated to obtain MOF derivatives, which partially inherit the properties of MOFs. Most MOF derivatives are generated by controlled annealing of specific MOF precursors, and both the choices of the precursor and the reaction conditions are critical. Typically, annealing in air results in complete decomposition of the organic ligands, thereby forming porous metal oxides.^148^ In contrast, inert atmospheres can convert ligands into porous carbons,^206^ which in turn yield metals/carbons,^207^ depending on the properties of MOF precursors. It needs to be mentioned that the pyrolysis conditions are crucial in determining the composition of the resulting porous carbon hybrids. Tailoring the pore structure of MOF derivatives can be achieved by means of improvements in the structure, morphology, and fabrication strategies of MOF precursors. The pore size distribution and specific surface area are highly correlated, and they are regulated by pyrolysis temperature, chemical activation, template strategy, and microstructural design. The temperature of pyrolysis plays the most critical role in tailoring the pore structure. Higher temperature favors the decomposition of MOFs to produce ordered graphitic carbon with high conductivity, while the original pore structure may collapse and metal compounds will be severely aggregated, resulting in a low specific surface area.^208^ Chemical activation (*e.g.*, KOH activation) can fabricate activated porous carbons to promote the specific surface area and porosity.^209^ Templates can be used to tailor the macroporous structure of MOF single crystals.^210^ After template removal, high temperature annealing, and removal of residual metal species, macropores were achieved through size tailoring in MOF-derived carbons.^211^ Ordered macropores can provide abundant mass transfer channels for EES.^212^ The size design of the microstructure of MOFs provides an idea for indirectly tuning the pore structure of MOF derivatives. On the one hand, the microscopic size of MOF precursors is carefully designed before transformation.^213^ On the other hand, the design of specific dimensions is achieved during the conversion of MOFs to derivatives.^214^

#### 6.2.2. Vacancy engineering

Among various defects, vacancies play critical roles in determining the physical/chemical properties of materials.^215,216^ Vacancies can be created in crystalline materials by moving atoms at random positions or removing them from the lattice. In MOFs and their derivatives, the generation of vacancies can be controlled by tailoring the structure of MOFs and regulating the transformation conditions of derivatives, which can generate different physical/chemical properties through breaking and reforming of bonds, lattice distortion, electronic compensation, electron localization, and the gap states at the Fermi level.^217,218^ The vacancies unsaturate some of the atoms in the ordered structure, thereby providing potential active sites for electrochemical reactions. By tuning the atomic charges around the vacancy sites, the electronic structure of the material can be fine-tuned. Meanwhile, vacancies change the porosity of the material.^219^ Therefore, precisely tailoring vacancies can increase the reactivity and accessibility of active sites. Recently, vacancy engineering of MOFs and their derivatives has attracted considerable interest.^220^ Generally, vacancy engineering is divided into coordinatively unsaturated metal sites and missing linkers in pristine MOFs, as well as cation vacancies, anion vacancies and carbon vacancies in MOF derivatives.

Metal centers in MOFs promise to provide potential active sites; however, fully coordinated metal ions have no available adsorption sites.^221^ Therefore, the unsaturated coordination of metal ions tends to expose more active sites.^222^ Based on the high designability of MOFs, the introduction of controllably missing linkers into pristine MOFs can alter their electronic structures without losing porosity and crystallinity.^223^ After the introduction of the missing linker, new electronic states are generated near the Fermi level, and the optimized electronic structure enhances the conductivity.^224^

The introduction of cation vacancies also has an important effect on the electrochemical properties of the materials. Metal vacancies have unusual properties due to their diverse electron and orbital distributions.^225^ The presence of metal vacancies tunes the electronic structure of the surface and increases the valence state of the nearby metal centers, thereby greatly enhancing the electrochemical activity. However, the high formation energy of metal vacancies leads to the difficulty of constructing metal vacancies. Therefore, some auxiliary strategies have been proposed for the formation of metal vacancies.^226^ Anion vacancies can induce unsaturated coordination sites to further optimize the local electronic structure and electronic environment.^227^ Owing to the low formation energy of oxygen vacancies, they have received extensive attention for application in electrode materials.^228^ It has been reported that oxygen vacancies can act as donors to enhance the conductivity of metal oxides and provide active sites for electrochemical reactions.^229^ In addition, other anion vacancies, such as sulfur vacancies and nitrogen vacancies, were also investigated.^230^ In fact, anion vacancies are more common in MOF-derived metal compounds and show enhanced energy storage properties.^231,232^ The vacancies in carbon structures largely affect their physical/chemical properties.^233^ The vacancies introduced in the carbon framework can directly serve as potential active sites to overcome the capacity limitation.^234^ The C–C sp^3^ defects enable more efficient diffusion pathways for ions,^235^ while vacancies and edge sites increase the capacity by adsorbing metal ions.^236^ In addition, heteroatom doping in the carbon structure can improve the ion storage performance by optimizing the electronic structure and forming vacancies.^237,238^

#### 6.2.3. Heterostructure engineering

To realize the multifunctionality of MOFs and their derivatives, the formation of composites by integrating different materials is expected to obtain desirable functionalities in a single system. This design strategy has been reported for electrode materials for MIBs and SCs.^239^ Although this component design strategy can achieve a reasonable combination of various properties, the rough interfacial coupling between the various components can easily lead to suboptimal reaction kinetics and high charge transfer resistance. Therefore, heterostructure engineering has been proposed to provide optimized schemes for atomic-scale coupling between different materials, enabling effective combinations of the physical/chemical properties of different components, and even yielding unique interfacial properties. In fact, the concept of heterostructures was first proposed in the field of semiconductor physics, not energy storage. As a broad definition, heterostructures are composed of more than two types of materials and exhibit indistinct interfaces and complex shapes.^240^ Unlike composites, heterostructures cause charges to redistribute around the heterointerface. Due to the multiple synergistic effects of heterostructures, most heterostructure electrodes exhibit higher electrochemical activity than their single-component electrodes. Heterostructures have fine band structures and small band gaps, providing excellent electrical conductivity. Due to the internal electric field present at the heterointerface, the electron/ion diffusion is accelerated and the driving force for charge transfer is enhanced, thereby improving the electrochemical reaction kinetics. Interactions between multiple phases in heterostructures enhance structural stability for more durable electrode materials. In addition, there are more active sites in the heterostructure, thereby improving the reversible capacity of the electrode material.^241^

Although heterostructures of pristine MOFs have been constructed,^242,243^ they are usually not directly used for EES due to their low conductivity and stability. An efficient way is to convert them into derivatives. Well-designed precursors are expected to obtain heterostructures after thermal or chemical transformation to achieve more stable structures and better electrochemical performance. Liu *et al.* synthesized a Ni-doped Co–Co_2_N heterostructure based on ZIF-L and provided a specific capacity of 361.93 C g^−1^.^244^ The full cell achieved an energy density of 20.4 W h kg^−1^ at a power density of 9.85 kW kg^−1^. Xiao and co-workers constructed In_2_Se_3_–CoIn_2_–CoSe_2_ heterostructures by selenization of In-MOF@ZIF-67.^245^ The synergistically enhanced Na^+^ diffusion and electrical conductivity show excellent reversible sodium storage capacity. The core–shell Bi_2_S_3_@Co_9_S_8_ heterostructure is derived from a two-step transformation of BiOBr@ZIF-67.^246^ The hollow heterostructures enhance the conductivity and reaction kinetics, thus exhibiting remarkable lithium and sodium storage properties. Anatase–rutile heterostructures with abundant oxygen vacancies were prepared by *in situ* topological conversion of NH_2_-MIL-125 (Ti).^247^ Surface/bulk oxygen vacancies and regulated C–N bonds accelerate electron/ion transport and improve the pseudocapacitive behavior of the electrodes. Theoretical calculations confirm that the introduction of an anatase–rutile heterostructure reduces the migration energy barriers of Na^+^ and Li^+^. Lou's group constructed a Mn_2_O_3_–ZnMn_2_O_4_ hollow heterostructure that provides abundant active sites and robust stability for Zn^2+^ storage.^248^ Therefore, designing advanced heterostructures will provide great opportunities for high-performance energy storage systems.

To date, heterostructures have been reported for SCs and MIBs.^249–251^ MIHCs are hybrid energy storage systems based on SCs and MIBs. Developing anodes with enhanced kinetics to match the fast kinetics of cathodes or increasing the capacity of cathodes will improve the overall performance of MIHC devices. Therefore, building advanced heterostructures in electrode materials for MIHCs will provide unparalleled performance for such hybrid energy storage systems.

#### 6.2.4. Nanostructure designs

To promote the structural stability and electrochemical activity of electrode materials, the design of nanostructures has attracted considerable attention. These nanoarchitectures present highly structurally related advantages, which stem from the high surface area, rational pore channels/voids, robust structures, *etc.*^252^ Among them, the high surface area provides a platform for surface-related processes, especially the insertion/deintercalation of ions and surface redox reactions.^253^ An appropriate pore structure and high pore volume are crucial for loading guests, facilitating mass transport, and accommodating the volume changes of active species.^254^ Unique channels and cavities also show potential applications. The fine and rational design of nanostructures is expected to endow materials with the required functionality for efficient energy storage devices.^255^ The most striking feature of MOFs is the designable framework formed by modular assembly. Furthermore, MOFs can be easily transformed into functional nanomaterials under thermal or chemical conditions. In addition to using simple MOFs as precursors, the fine design and combination of MOFs can be used to construct well-designed advanced nanoarchitectures and provide optimized performance, providing a potential scheme for advanced EES.^256^ Herein, the design of some representative nanostructures is discussed to construct novel high-performance electrode materials for MIHCs.

The hollow structures obtained from MOFs have open channels, tunable voids, and well-defined active sites.^257^ The volume collapse of MOFs during high-temperature calcination creates a large number of internal voids within the structure. The porous hollow structure produced in this way can effectively enhance the electron/ion diffusion kinetics. In addition, the hollow structure not only facilitates an effective contact between the active material and the electrolyte, but also alleviates the dramatic volume expansion of the active material during electrochemical cycling.^258^ Typically, Ni-MOFs with unique hierarchical hollow structures are carbonized and oxidized to obtain graphene shell-encapsulated hierarchical NiO/Ni.^259^ The well-designed hierarchical hollow sphere structure alleviates the dramatic volume expansion of NiO during cycling and provides high electrical conductivity, thus showing superior lithium and sodium storage capacity.

By ingeniously constructing shell-containing structures, nanoarchitectures can be endowed with special functionality and stability. Among them, the core–shell structure is the most representative.^260^ In the core–shell structure, the existence of the shell layer well protects the inner core and provides a stable environment for electrochemical reactions. Meanwhile, the synergy between the core–shell components achieves the complementarity of the properties of each component, such as facilitating electron transfer and increasing electrical conductivity. In addition, core–shell structures can serve as templates for designing hollow structures. In a typical example, a ZnS–Sb_2_S_3_@C core–double shell structure was synthesized based on ZIF-8.^261^ In particular, the carbon shell acts as a protective layer to form a rigid structure to accommodate volume changes and improve electronic conductivity. This core–double shell structure facilitates the penetration of the electrolyte, thereby reducing the Na^+^ diffusion distance to improve the kinetics. Yolk–shell structures are a special class of core–shell structures that are characterized by the retention of a gap between the core and the shell. The use of well-designed yolk–shell structures is an effective strategy to realize robust electrode materials. The yolk–shell structure has a core and an outer shell, wherein the outer shell protects the inner yolk and increases structural stability.^262^ The stable yolk–shell structure provides sufficient void space to accommodate volume changes and helps relieve stress caused by volume expansion of active substances during cycling. Additionally, functionalized active sites doped in the outer shell can provide enhanced electrochemical activity and conductivity. For instance, by controlling the morphological evolution of MIL-53 (Fe) assisted by microwaves and adjusting the irradiation time, a mesoporous yolk–shell Fe_2_O_3_ nanostructure was obtained and provided excellent lithium storage performance.^263^

Besides single-shelled and few-shelled structures, multi-shelled structures with a larger void volume and high surface area-to-volume ratio offer great potential for EES. Among them, a large amount of void space is well adapted for the volume change in the reaction, and a relatively high proportion of active components improves the volumetric energy density.^264^ However, their controllable design and construction face greater challenges.

## 7. Conclusions and prospects

To summarize, we have witnessed the rapid development of MIHCs in the past decade. However, the kinetic imbalance between their cathodes and anodes and the lack of high-performance electrode materials are the major challenge. The unique properties of MOFs and MOF derivatives have led to significant progress in MIHCs from both fundamental and applied perspectives. This review attempts to propose advances in MOFs and MOF derivatives that remain to be discovered to meet the requirements of practical MIHC applications. Specifically, we first briefly describe the construction of MIHCs and their charge storage mechanisms, with a special focus on electrode and cell configurations, as well as electrolytes. The application of MOFs and their derivatives as multifunctional platforms in MIHCs is then revealed from the perspectives of pristine MOFs, MOF composites, and MOF derivatives. Furthermore, in order to improve electrode materials that are an important part of MIHCs, several optimization strategies are proposed for constructing advanced MOFs/MOF derivatives and using them as high-performance electrode materials. On the one hand, MIHCs integrate the high energy density of MIBs with the high power density and excellent cycling capability of SCs, and are expected to be applied in large-scale energy storage in the future. On the other hand, since MOFs and their derivatives have made great progress in recent years, they have been actively explored for EES and even extended to emerging MIHCs.

As key components of MOFs, organic ligands have important effects on the structure and electrochemical performance of MOFs or MOF derivatives. For pristine MOFs, the low conductivity of most pristine MOFs is the result of insulating and redox-inactive bridging ligands. Conductive MOFs achieve intrinsic electronic conductivity, while retaining the advantages of the metal–ligand coordination and crystalline porous structure of MOFs. Current research mainly employs transition metals and conjugated organic ligands to construct 2D π–d conjugated porous frameworks.^265^ Among them, 2, 3, 6, 7, 10, 11-hexaiminotriphenylene (HITP), HHTP and HAB are the most commonly used ligands to synthesize conductive MOFs and fabricate conjugated porous structures. On the other hand, amino and sulfonate groups are commonly used functional groups that facilitate the proton transport pathway to enhance proton conductivity.^266^ The geometry of the conjugated ligands generally determines the topology and pore structure of conductive MOFs. Furthermore, changes in conjugated ligands or metal nodes may directly affect the layer stacking mode of conductive MOFs.^267^ Besides conductive MOFs, PBAs have received considerable attention due to their open frameworks. N-coordinated cations and C-coordinated cations in PBAs exist in open frameworks bridged by cyanide groups. The capacity of the electrode is contributed by the electrochemical redox reaction of transition metal ions. Both N-coordinated and C-coordinated transition metal ions are electrochemically active. Moreover, compositional control of transition metal ions can be achieved in PBAs to optimize the charge storage behavior of PBAs.^268^ For MOF derivatives, the most common conversion strategy for MOF precursors is thermal treatment. The products obtained from organic ligands at a high temperature depend on the gas atmosphere during pyrolysis. In an air atmosphere, the organic ligands are oxidized to CO_2_ and removed. In an inert atmosphere, the organic ligands were decomposed into carbon frameworks and the porous structure was preserved.^269^ Some heteroatoms in the ligands are doped into the carbon framework. The abundant functional groups formed by heteroatom doping provide additional sites for ion storage and further enhance the conductivity and wettability of the material. ZIFs and MILs are the most classical MOF precursors. Among the subclasses of ZIFs, ZIF-8, ZIF-67 and ZIF-L have been extensively studied with 2-methylimidazole as their ligand.^270^ ZIF-8 and ZIF-67 are usually synthesized in methanol, and they have the same dodecahedral structure and micropore-dominated pore structure. In the aqueous system, 2D leaf-like ZIF-L is obtained. The abundant nitrogen source in 2-methylimidazole provides nitrogen doping for better electrochemical performance after pyrolysis. A common ligand in MILs is terephthalic acid. Terephthalic acid provides an oxygen source for the formation of metal oxides after pyrolysis.^271^ To obtain nitrogen-doped carbon, 2-aminoterephthalic acid is commonly used rather than terephthalic acid. Different solvents or reaction temperatures have important effects on the structure and exposed facets of MILs, which correlate with their properties.^272^

High-performance MIHCs need to provide satisfactory energy density, power density, and long cycle life. Among them, the matching of cathode and anode materials in capacity and kinetics is the key factor. The slow kinetics of battery-type materials results in unsatisfactory power densities for MIHC devices. Therefore, accelerating the ion transport rate is the key to improving the kinetics of battery-type electrodes. Conductive MOFs have high electrical conductivity and a simple fabrication process, and the highly ordered nanopores can reduce ion diffusion paths, thereby increasing power density.^267^ With the rational design of metal ions and organic ligands, conductive MOFs provide more abundant active sites to enhance capacity. The electrical conductivity of PBAs is unsatisfactory. The hybridization of PBAs with various conductive substrates is one of the effective strategies to overcome this problem.^273^ Rational metal substitution, construction of core–shell structures, and morphological design can improve the electrochemical performance of PBAs.^268^ Among them, metal substitution provides atomic-level modulation and fundamentally optimizes the electrochemical performance of PBAs. On the other hand, MOF derivatives possess diverse nanostructures, tunable configurations, uniformly distributed pores, and multidimensional morphologies, which can be controlled by tuning the metal centers and organic ligands of MOF precursors. Their performance is mainly enhanced by the modulation of the pore structure and nanostructure, as well as exposing more active sites.^274^ The pyrolysis of pristine MOFs in an inert gas atmosphere to produce MOF derivatives is the most widespread method. The formation of the carbon framework imparts different levels of electrical conductivity to the derivative. Depending on the pyrolysis temperature and the structure of the precursors, the pyrolysis process may also lead to the formation of hierarchical and interconnected porous structures to accelerate ion transport rates. On the other hand, capacitor-type materials with high capacity are beneficial to improve the energy density of MIHC devices. The performance of capacitor-type materials originates from charge adsorption and transport at the electrode/electrolyte interface or reactions at redox sites, which are determined by the intrinsic properties and structural design of electrode materials, including electrical conductivity, pore structure, specific surface area, and active sites. Capacitor-type materials are usually based on carbon materials and pseudocapacitive materials. For carbon materials, the pore structure needs to be optimized to ensure the compatibility of carbon materials with electrolyte ions, which can be further achieved by tuning the pore structure of MOF precursors.^275^ In addition, the tuning of microstructures, design of nanostructures, and heteroatom doping are included. The modulation of the microstructure is achieved by tuning the composition and synthesis conditions of MOFs, and the design of nanostructures requires creative improvements to MOF precursors, such as the construction of complex structures. The heteroatom-containing ligands in MOFs can form heteroatom-doped carbon after pyrolysis in an inert atmosphere, which requires targeted selection of ligands or modification of ligands. In addition, guest molecules are also permitted to provide sources of heteroatoms. Besides the optimization of carbon materials, the development of pseudocapacitive materials with high specific capacitance is also an effective way to improve the energy density of MIHCs. Since the pseudocapacitance is based on the reversible surface faradaic reaction to store charges rather than ion adsorption/desorption. The metal nodes and organic linkers of pristine MOFs significantly affect the pseudocapacitive performance when they participate in redox reactions.^276^ Meanwhile, the stability of MOFs is highly correlated with the coordination bond strength between metal nodes and ligands, where the metal–ligand coordination bond strength is usually positively correlated with the metal ion charge and negatively correlated with the metal ion radius. For MOF derivatives, it is necessary to provide a larger specific surface area, highly exposed active sites, and sufficient electron/ion transport channels, which places high demands on the structural design of MOF precursors, including the design of pore structures and nanostructures, as well as modulation of components.

**Fig. 14:**
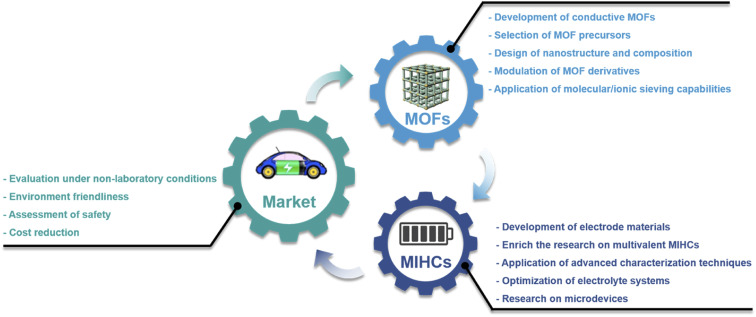
Challenges of MOFs and their derivatives for MIHCs.

However, these developments come with challenges and opportunities. When we focus on MOFs and their derivatives to shine in MIHCs, we still need to pay attention to the current shortcomings and clarify future research directions. Here, possible future research directions are proposed from the perspective of MOFs and their derivatives and MIHCs (Fig. 14). MOFs offer diverse structures and functionalities due to the high designability of organic linkers and the selectivity of metal nodes. In addition, MOF derivatives have also been rapidly developed to meet the multiple requirements of energy storage devices. In the following, from the perspective of MOFs/MOF derivatives and combined with MIHCs, feasible research directions in the future are proposed.

(i) Although conductive MOFs have received considerable attention as electrodes for MIHCs due to their high conductivity and tunable pore size, the controllable synthesis of conductive MOFs with targeted structures remains elusive, resulting in unsatisfactory properties (*e.g.*, moderate specific surface area and poor crystallinity). Significant progress in optimizing synthetic strategies for conductive MOFs would be of great value. Meanwhile, for a comprehensive understanding of the electron transport mechanism of conductive MOFs, theoretical calculations can provide a reasonable platform for understanding the relationship between the structure and electronic properties of MOFs. Furthermore, the reported conductive MOFs are mainly concentrated on 2D structures. However, their electrochemical performance is limited by their moderate specific surface area and 1D-like channel for ion swapping. This can be countered by designing 3D conductive MOFs with a high specific surface area and abundant ion channels, which facilitate the transport of ions in all directions, leading to efficient cation–anion swapping.^277,278^

(ii) Most of the precursors used to prepare MOF derivatives are derived from the ZIF subclass, ZIF-8 and ZIF-67 are the two most representative precursors. Despite the high stability, ideal metal sources, and abundant nitrogen sources of these two classical MOFs, their microporous structures lead to a singulated pore structure in the derivatives, and the micropores may lead to suboptimal active site utilization and restricted mass transfer. The pore structure is a typical property of MOFs, and the important role of the pore structure in MIHCs has been highlighted in previous chapters. Some other MOFs, for example, MIL-101, have a larger specific surface area than ZIF-8 and ZIF-67, and a mesoporous cage (2–3 nm) structure. In addition, it is necessary to develop other MOFs as precursors to enrich the study of MOF derivatives.

(iii) Among MOF-derived carbons, some heteroatom-doped carbons have attracted considerable attention due to the introduction of more active sites and defects by the doping of heteroatoms. More importantly, heteroatom doping leads to enlarged interlayer spacing in carbon materials, as well as an altered electronic structure. These factors are critical for increasing the capacity of carbon materials to match the high capacity of battery-type materials. However, single heteroatom doping has been unable to meet the current requirements. In recent years, dual-atom doping has gradually become a research hotspot. In order to make up for the deficiency of single heteroatoms and pursue a stronger synergistic effect, tri-atom or even multi-atom doping is expected to gain more attention and research in the future. However, it would lead to difficulty in understanding the interaction mechanism between dopants and substrate materials. Therefore, better insights into their roles can be obtained by combining advanced characterization techniques and theoretical calculations. On the other hand, the framework structure of some MOFs may collapse after high-temperature pyrolysis, and the original pore structure cannot be well preserved, which is due to the high pyrolysis temperature and their poor stability. The disruption of the framework structure and pore structure may lead to unsatisfactory electrochemical performance. It should be noted that additional thermal treatment of MOFs consumes more energy for material fabrication and increases the already high cost of MOFs. In this case, it is unwise to blindly use MOF-derived carbon instead of directly using low-cost carbon materials. To deal with this problem, it is feasible to select MOFs with good thermal stability and explore the most suitable pyrolysis temperature.

(iv) For MOF-derived metal compounds or metal compound/carbons, they are commonly used as battery-type electrodes. During continuous cycling, metal compounds are prone to volume expansion, structural damage, or lattice distortion. Heteroatom doping in metal compounds can achieve better stability and electrochemical performance by adjusting the electronic structure, and can also suppress the lattice distortion of the material during the charge/discharge process, and improve the ion diffusion rate, thereby showing better rate performance. Heteroatom doping in MOF-derived metal compounds is expected to be achieved by bimetallic MOFs or by introducing additional metal sources. On the other hand, battery-type materials based on intercalation reactions suffer from large volume expansion during the intercalation/deintercalation of metal ions, resulting in unsatisfactory cycling stability. Utilizing uniformly distributed metal nodes of MOFs to achieve nanoscale active materials is a feasible approach to alleviate volume expansion. In addition, the introduction of sufficient void space and a conductive matrix into conversion-type and alloy-type materials is an effective way to mitigate volume expansion and improve low electrical conductivity. Therefore, it is necessary to carefully design the nanostructure and composition of MOF precursors to fabricate advanced nanoarchitectures for MIHCs.

(v) MOFs provide efficient molecular/ion sieving capabilities. As well-known molecular sieves, MOFs have long been used for molecular-scale gas adsorption/separation and the separation of soluble mediators. MOFs with a tunable porosity, highly ordered pore structure and large surface area are also candidates for use as ionic sieves. By appropriately adjusting the pore size, target ions can be separated from the solution. Therefore, MOFs have unique size effects on a variety of molecules and ions. Theoretically, the ordered pore structure of MOFs can be precisely controlled by judiciously combining inorganic and organic units. The molecular/ionic sieving ability and unique size effect based on MOFs motivate the development of advanced separators using MOFs with specific channel sizes. Although the current reports on MOF-based separators applied to MIHCs are very limited, the inherent properties of MOFs determine that they will surely occupy a prominent position in separators and their modification.

(vi) The introduction of binders may lead to the occupation of pore space or blockage of pore channels of MOFs or MOF derivatives, thereby reducing the overall performance of the material. The growth of MOFs on the substrate can adjust the growth direction of MOFs.^279^ Meanwhile, the intrinsic resistance and charge transfer resistance of the electrode/electrolyte interface of binder-free highly ordered electrodes are significantly lower than those of powder electrodes. The utilization of pores can be significantly improved by the fabricated highly uniformly oriented electrodes, thereby improving the electrochemical performance.

Although MOFs and their derivatives have made great progress in the application of MIHCs, there are still some unclear mechanisms and unsolved problems in MIHCs. In order to promote the longer-term development of MOFs/MOF derivatives in MIHCs and the feasibility of MIHCs in practical applications, improvements and research can be made in the following aspects in the future.

(i) In monovalent MIHCs, Na^+^ and K^+^ have higher ionic radii than Li^+^, and these properties lead to sluggish kinetics and unsatisfactory cycling performance of the anode. For multivalent MIHCs, the fast diffusion of cations is hindered and the power density decreases due to the strong electrostatic interaction between the cations and the crystal structure of the electrodes. To address these difficulties, advanced electrode materials with porous structures and abundant active sites should be explored. In addition, nanonization, heteroatom doping, and structural design of materials are effective strategies to optimize electrodes. Nanonization produces fast diffusion kinetics by shortening ion transport distances. Heteroatom doping can tune the electronic structure of materials and improve electrical conductivity and electrochemical activity. The precise structural design can effectively alleviate the volume expansion of the active material during charging and discharging to improve the cycle stability. In fact, the above optimization strategies can be easily realized by MOFs, which is mainly attributed to the designable metal nodes and organic linkers of MOFs. Furthermore, the development of novel anodes with large-channel ion transport is critical for MIHCs, and conductive MOFs can satisfy this condition.

(ii) Among multivalent metal ions, Ca^2+^, Mg^2+^ and Al^3+^ are also attractive due to their inherent advantages. On the one hand, they have relatively abundant reserves. On the other hand, their multi-electron redox properties are expected to achieve high energy densities. However, the reports of MIHCs based on Ca^2+^, Mg^2+^ and Al^3+^ are very limited. To our knowledge, the application of MOFs and their derivatives in these devices has not been reported, and future research can be expected to enrich this field.

(iii) The charge storage mechanisms and reaction kinetics in electrode materials need to be further understood. *In situ* characterization techniques, such as *in situ* XRD, *in situ* transmission electron microscopy (TEM), and *in situ* Raman, have been used to monitor the structure and phase transitions of electrodes in real time during charge and discharge. Among them, *in situ* XRD is widely used to monitor the phase transition of electrode materials in real time during cycling. *In situ* TEM provides an efficient method to monitor structural evolution during sample formation or electrochemical processes. Combined with technologies such as selected area electron diffraction (SAED) and electron energy loss spectroscopy (EELS), more abundant chemical information can be obtained. *In situ* Raman can reveal the evolution of the structural and electronic properties of electrode materials during charge and discharge. Due to the complementarity of *in situ* techniques, the use of multiple *in situ* techniques is beneficial to study electrochemical mechanisms from different perspectives. In addition, theoretical calculations enable a deeper understanding of electrochemical performance and guide the design of electrode materials.

(iv) From the perspective of electrolytes, the narrow potential window of aqueous electrolytes is difficult to support the long-term operation of MIHCs at high voltages, and the theoretical operating voltage is limited to 2 V. Nonaqueous electrolytes suffer from low ionic conductivity, high viscosity, high cost and high toxicity. Therefore, it is very important to develop inexpensive, safe and efficient electrolyte systems. A “water-in-salt” electrolyte overcomes the shortcomings of aqueous electrolytes by confining water molecules and preventing parasitic reactions to provide an extended voltage window to achieve high energy density.^280^ Appropriate redox additives can effectively improve device performance. In addition, a hybrid aqueous/nonaqueous electrolyte provides a new idea for broadening the study of electrolyte systems.^281^

(v) From the perspective of commercialization, the area/volume energy density is critical for device design. Electrodes with high mass loading and low electrolyte uptake are required to design MIHCs with a high area/volume performance. The amounts of inactive ingredients (*e.g.*, conductive agents, binders, and electrolytes) vary significantly between laboratory and commercial devices. In commercial devices, compact MIHCs are designed at low cost by reducing the electrolyte volume in the device as much as possible. In high active material loading or flexible devices, the active material tends to detach from the substrate, resulting in a shortened cycle life. Therefore, there is a need to develop advanced electrode fabrication methods and current collectors. In addition, the fabrication cost is a key factor that must be considered, and it is necessary to prepare electrode materials and electrolytes through low-cost, green fabrication processes.

At present, among the numerous types of MIHCs, the energy density of commercialized LIHCs still cannot meet the requirements of energy storage in the future, while other types of MIHCs are still in the early stage of academic validation. Therefore, further improving the performance metrics of MIHCs remains a huge academic and industrial challenge. Due to the highly tunable metal nodes and organic linkers, MOFs and their derivatives exhibit designable structures and chemical compositions. MOFs and their derivatives as multifunctional platforms have provided great opportunities to promote the development of MIHCs. With the discovery of advanced fabrication processes and characterization techniques, combined with theoretical calculations to guide the design of such materials, MOFs and their derivatives will make significant contributions to advancing the development of MIHCs. Driven by the huge market demand, MIHCs will also gradually achieve breakthroughs. With further improvement in their performance and reduction in cost, MIHCs will show great potential in meeting high energy/power market demands. Therefore, we hope that this review can inspire and guide research in this emerging field and contribute to the design/optimization of MOF-based materials and their diverse applications in MIHCs.

## Author contributions

The manuscript was written through contributions of all authors. All authors have given approval to the final version of the manuscript.

## Conflicts of interest

There are no conflicts to declare.

## Supplementary Material
